# Chronic Sympathetic Hyperactivity Triggers Electrophysiological Remodeling and Disrupts Excitation-Contraction Coupling in Heart

**DOI:** 10.1038/s41598-020-64949-7

**Published:** 2020-05-14

**Authors:** Humberto C. Joca, Artur Santos‐Miranda, Julliane V. Joviano-Santos, Rebeca P. M. Maia-Joca, Patricia C. Brum, George S. B. Williams, Jader S. Cruz

**Affiliations:** 1 Department of Biochemistry and Immunology, Institute of Biological Sciences, Federal University of Minas Gerais, Belo Horizonte, MG Brazil; 2 Center for Biomedical Engineering and Technology, University of Maryland School of Medicine, Baltimore, Maryland USA; 3 Department of Biophysics, Universidade Federal de Sao Paulo, Sao Paulo, SP Brazil; 4 School of Physical Education and Sport, University of São Paulo, São Paulo, SP Brazil

**Keywords:** Heart failure, Ion transport, Computational biophysics, Cardiovascular biology, Calcium signalling, Ion channel signalling

## Abstract

The sympathetic nervous system is essential for maintenance of cardiac function via activation of post-junctional adrenergic receptors. Prolonged adrenergic receptor activation, however, has deleterious long-term effects leading to hypertrophy and the development of heart failure. Here we investigate the effect of chronic adrenergic receptors activation on excitation-contraction coupling (ECC) in ventricular cardiomyocytes from a previously characterized mouse model of chronic sympathetic hyperactivity, which are genetically deficient in the adrenoceptor α2A and α2C genes (ARDKO). When compared to wild-type (WT) cardiomyocytes, ARDKO displayed reduced fractional shortening (~33%) and slower relaxation (~20%). Furthermore, ARDKO cells exhibited several electrophysiological changes such as action potential (AP) prolongation (~50%), reduced L-type calcium channel (LCC) current (~33%), reduced outward potassium (K^+^) currents (~30%), and increased sodium/calcium exchanger (NCX) activity (~52%). Consistent with reduced contractility and calcium (Ca^2+^) currents, the cytosolic Ca^2+^ ([Ca^2+^]_i_) transient from ARDKO animals was smaller and decayed slower. Importantly, no changes were observed in membrane resting potential, AP amplitude, or the inward K^+^ current. Finally, we modified our existing cardiac ECC computational model to account for changes in the ARDKO heart. Simulations suggest that cellular changes in the ARDKO heart resulted in variable and dyssynchronous Ca^2+^-induced Ca^2+^ release therefore altering [Ca^2+^]_i_ transient dynamics and reducing force generation. In conclusion, chronic sympathetic hyperactivity impairs ECC by changing the density of several ionic currents (and thus AP repolarization) causing altered Ca^2+^ dynamics and contractile activity. This demonstrates the important role of ECC remodeling in the cardiac dysfunction secondary to chronic sympathetic activity.

## Introduction

Activation of the sympathetic nervous system (SNS) exerts several effects on the cardiovascular system. These include accelerating heart rate, increasing cardiac contractility, reducing venous capacity, and promoting vasoconstriction of resistance arteries^1^. Coordination of sympathetic activity through cardiovascular reflexes enables accurate, short and long term control of blood flow and pressure^2^. Furthermore, a physiological increase in sympathetic activity is a determinant for cardiovascular responsiveness to exercise or other stress stimuli (e.g., fight-or-flight response). In the heart, SNS effects are mainly through stimulation of β-adrenergic receptors (β-AR)^3^.

The decline in the cardiac contractile capacity after an acute myocardial infarction or chronic pressure overload^4^ triggers the activation of compensatory mechanisms such as renin-angiotensin-aldosterone system (RAAS), the cytokine system, and the SNS^5^. Regarding SNS, continuous or excessive stimulation of β-AR in the myocardium might trigger several deleterious effects. For example, hyper-activation of β1AR receptors can drive cardiomyocyte hypertrophy, mitogenesis of cardiac fibroblasts, and even apoptosis^4,5^. Such effects arising from increased sympathetic tone are described as critical factors for the progressive development of various cardiovascular diseases including heart failure (HF)^4,6^.

There is strong evidence supporting the role of increased sympathetic tone in the progression of several cardiac dysfunctions. Less understood, however, is the role of increased sympathetic tone in the modification, conduction, and propagation of the electrical stimulus throughout the cardiac muscle. Additionally, details regarding increased sympathetic tone on cardiac ion channel function, calcium handling, and cell contraction are poorly elucidated and existing experimental models are mostly unsuitable for the study of the role of SNS in the observed deleterious cardiovascular diseases^6^.

The present study explored the role of chronic sympathetic hyperactivity (CSH) in the remodeling of the excitation-contraction coupling (ECC). We utilized a genetic model of sympathetic hyperactivity created by knocking out α2A and α2C adrenergic receptors. While α2 adrenergic receptors are absent in the cardiac myocytes, they provide inhibitory feedback in adrenergic synapses and are an essential factor for the control of sympathetic tone. Hein *et al*., 1999^7^ showed that the presynaptic α2A and α2C receptors modulate release of neurotransmitters in the SNS terminals. The removal of these receptors leads to sympathetic hyperactivity and increased plasmatic norepinephrine, causing mice to be prone to developing cardiac hypertrophy, exhibiting reduced physical capacity, impaired cardiac contractility, changes in cardiomyocyte ultrastructure, and higher mortality with age progression after 5 months^6,7^. Due to these characteristics, the adrenergic receptor double knock-out (ARDKO) animal model is well suited for the study of the role of sympathetic tonus increase in development and progression of cardiac disease.

Using this ARDKO animal model we investigated the influence of CSH on cardiac ECC and found that CSH induces an electrochemical remodeling in ventricular cardiomyocytes (VCMs) which included action potential (AP) prolongation, altered ion currents, and impaired cellular contractility and Ca^2+^ handling.

## Methods

### Animals

All experimental procedures were conducted according to guidelines from both the Brazilian Society of Laboratory Animal Science (SBCAL) and the United States’ National Institutes of Health (NIH) Animal Care Guide. The project was approved to the Animal Use Ethics Committee of the Federal University of Minas Gerais (CEUA - UFMG) without restriction to the proposed procedures (Protocol No. 340/2013). Two animal groups were used, the ARDKO (males, 3 and 7 months old) in C57BL6/J genetic background, and wild-type (WT) controls (males, 3 and 7 months old). At the age of seven months, ARDKO mice had cardiac dysfunction associated with exercise intolerance and a higher mortality rate^6,8^. The animals were obtained from the School of Physical Education and Sport, University of São Paulo, São Paulo, SP, Brazil.

For indirect measurement of heart rate (HR) and mean arterial pressure (MAP) in WT and ARDKO animals (7 months old), we used a rodent plethysmograph (Kent Scientific Corporation, Torrington, CT). The mice were contained using a heated acrylic chamber and the body temperature was maintained at 35 ± 2 °C with constant monitoring by an infrared thermometer. The animals’ tail was then fitted to a rubber cuff that was adapted to the proximal region and connected to the sphygmomanometer to inflate and deflate automatically at fixed intervals of 50 seconds. Near the cuff was attached a pulse transducer that captured the signals and recorded them on a computer. The measurements only started after 5 minutes of acclimatization. MAP and HR were calculated automatically by the software from the data obtained from 3 consecutive measurements.

### Cardiac myocytes isolation

To obtain VCMs, the animals were euthanized by decapitation and hearts were carefully removed via thoracotomy, cannulated through the aortic trunk, and then mounted in a Langendorff constant pressure system, where they were perfused in a retrograde flow system^9^. In this process, hearts were perfused with Ca^2+^-free, cell-isolation buffer (CIB), containing (in mM): 130 NaCl, 5.4 KCl, 0.5 MgCl_2_, 0.33 NaH_2_PO_4_, 22 glucose, 25 HEPES, and 0.4 EGTA (pH 7.4), maintained at 37 °C for 3 min to clean the heart. After this process, the Ca^2+^-free CIB solution is replaced by CIB (without EGTA) containing 0.3 mM CaCl_2_, 1 mg ml^−1^ collagenase type II, 0.06 mg ml^−1^ protease type XXIII, and 0.06 mg ml^−1^ trypsin, which was perfused for 3–5 minutes. The heart was then removed from the cannula and the ventricles were separated, cut into small pieces, and then subjected to an additional 8 min of digestion at 37 °C, with CIB supplemented with collagenase, trypsin, and protease. After these two steps of enzymatic digestion, the cells were isolated from the tissue by light agitation using a glass pipette. This solution containing the isolated cells was then filtered, centrifuged at 100 g for 30 sec, resuspended in CIB solution containing 1.2 mM CaCl_2_ and 2 mg/ml bovine serum albumin (BSA), and maintained in this solution for 10 min at 37 °C. Finally, the cells were centrifuged again (100 g for 30 s) and maintained in a Tyrode’s solution with the following composition (in mM: 140 NaCl, 5.4 KCl, 0.5 MgCl_2_, 0.33 NaH_2_PO_4_, 11 Glucose, 5 HEPES and 1.8 CaCl_2_, pH adjusted to 7.4). VCMs were used for experiments within 6 hours of isolation. VCMs with rounded edges and other visible signals of membrane damage or VCMs not responsive to electrical stimulation were not used for experiments.

### Cellular contractility and electrophysiology

The cellular contractility was measured by overall cell shortening using an edge detection system with high-speed camera (200 Hz, IonOptix, Milton, USA) at room temperature (i.e., 20–25 °C). Cells were placed in a perfusion chamber filled with Tyrode’s solution (see above) mounted on an inverted microscope (Nikon TS-100, Nikon Instruments Inc, Japan) and connected to two parallel metal electrodes immersed in the solution to provide the electric field stimulation (Grass Instruments, 40 V, 4 ms). The stimulation frequency ranged from 1 to 3 Hz depending on the experiment to be performed. A separated subset of cells was exposed to isoproterenol (ISO, 100 nM) prior to the contractility experiments.

The records of the APs and ionic currents was performed using the patch clamp amplifier EPC-10 (HEKA Elektronik, Germany) and recorded on a computer via the PACTHMASTER acquisition software (HEKA Elektronik, Germany) in the “Current clamp” and “Voltage clamp” modes. All experiments were performed at room temperature. To record the ion currents, the internal and external solution were specific for the current to be studied (see Supplemental Tables S1-S4). All cells were maintained at a holding potential of −80 mV when not stimulated by the protocols described in the Results. Only cells with high seal resistance (≥1GΩ) and low access resistance (≤10MΩ) were used for the electrophysiology experiments.

To trigger AP initiation (in current-clamp mode), the myocytes were stimulated with a brief depolarizing current (1 nA for 2 ms) and the changes in the membrane voltage was observed for 950 ms (at 1 Hz stimulation) and 320 ms (at 3 Hz). For the recording of NCX current, whole-cell membrane currents was measured using a voltage ramp ranging from −90 mV to +60 mV before and after exposing the cell with Ni^2+^ (5 mM) and using solutions designed to prevent contamination with Cl^–^currents^10^. The NCX current was obtained after the subtraction of those currents before and after the Ni^2+^ application, thus yielding only the Ni^2+^-sensitive membrane current component. For LCC currents, the cells were stimulated with square voltage steps (300 ms in duration) from −80 to +60 mV in 10 mV increments, every 10 seconds. For outward potassium currents, the protocol consisted of square voltage steps (3 seconds in duration) from −60 to +70 mV in 10 mV increments, every 10 seconds. The inward potassium currents were elicited with a square voltage steps (3 seconds in duration) from −120 to −50 mV in 10 mV increments, every 10 seconds. In a subset of LCC current recordings, cells were pre-treated with isoproterenol (ISO, 100 nM) before the measurements.

### Fluorescence microscopy

For confocal microscopy and [Ca^2+^]_i_ transient recording, VCMs were loaded with Fluo-4 acetoxymethyl (AM) for 30 minutes (20–25 °C) at a concentration of 5 μM in Tyrode’s solution. For NO and ROS measurements, the VCMs were loaded for 30 minutes using the DAF-FM (4-amino-5-methylamino-2 ‘, 7'-difluorofluorescein diacetate) or DCFH-DA (2’, 7’-dichlorofluorescein- diacetate) at concentrations of 10 and 3 μM, respectively. After the loading period, VCMs were then centrifuged and resuspended in Tyrode’s solution, where they remained for another 10 minutes allowing for de-esterification of the probes. The confocal images were acquired using an LSM 510 META confocal microscope (Carl Zeiss, Germany) from the Center for Acquisition and Image Processing of UFMG (CAPI - ICB - UFMG). VCMs were placed in a perfusion chamber filled with Tyrode’s solution and the probe was excited by an argon laser (488 nm). Emitted light was filtered through a long pass filter (505 nm) and collected by a photomultiplier tube. Similar to the cellular contractility experiments, an electrical stimulus with a frequency of 1 Hz was used to induce the [Ca^2+^]_i_ transients. The acquisition of the [Ca^2+^]_i_ signals was performed using the confocal in line-scan mode (1.84 ms/line) on middle portion of the myocyte avoiding the nuclei. Since the basal fluorescence (F_O_) was not different between groups, the changes in fluorescence (F) were normalized by the initial fluorescence (F_O_) and displayed as F/F_O_. For NO and ROS measurements, cellular fluorescence was measured using a Nikon Eclipse Ti microscope (Nikon, Japan). DAF and DCF (2’,7’-dichlorofluorescein; fluorescent form of DCFH-DA) were excited by a Xeon light source (Lambda DG4, Sutter Instrument, USA) filtered at 480/30 nm, and emitted light was filtered through a 535/40 nm band-pass filter before being collected by an EMCCD camera (Nikon, Japan). The acquisition parameters (e.g., camera gain, exposure time, etc) were kept the same throughout all the experiments. After subtraction of background fluorescence, the mean fluorescence intensity of each cell was measured. The DCFH oxidation to DCF is highly non-specific and responds to reactive species beyond just H_2_O_2_^11^. Due this fact, changes in DCF florescence are interpreted as changes in the cellular redox environment (global ROS production) rather than direct changes exclusively in H_2_O_2_ production.

### Protein expression

Protein concentration of the isolated VCMs (from individual hearts) was measured via a Lowry protein assay^12^. From each heart cell isolation procedure, a small fraction of the myocytes were separated to obtain the cell homogenate, which was centrifuged at 10,000 g for 30 min at 4 °C and the supernatant aliquoted and immediately frozen at −80 °C. The proteins were applied in equal amounts on polyacrylamide gel (10%) for the electrophoresis, then transferred to a nitrocellulose membrane. After the incubation with the specific antibodies from NCX (Abcam, ab6495) and GAPDH (Santa Cruz, SC-25778) at the recommended dilutions and a peroxidase conjugated to a secondary antibody, the signal was detected using the western blot detection system (ImageQuant LAS 4000, GE, USA). The images obtained were analyzed for protein levels using the IMAGE-J software. Since GAPDH is used for normalization, the data is presented both formats, raw density (arbitrary value) and normalized (NCX/GAPDH).

### Data analysis and simulations

The data in the graphs are presented as mean ± SD bars with a scatter plot of the individual data points. The number of cells/samples (n) for each data set is indicated. For the comparison of two groups, the unpaired Student’s t-test (parametric) or Mann-Whitney test (non-parametric) was used. For comparison of more than two groups, one-way or two-way ANOVA was used, followed by a Bonferroni multiple comparison test, when appropriate. The threshold for statistical significance was p ≤ 0.05 and reported p-values should be considered descriptive due to the exploratory nature of the study. The data collection and analysis were performed by non-blinded subjects. The sample size was not pre-defined and adjusted to include all the recording from the cells obtained from each experimental series.

The contractility parameters were obtained from routines preprogrammed in IonWizard software (IonOptix, USA). For each VCM, the average of five steady-state contractions (after 30 second stimulation train) was used for the calculation of the parameters for each cell. The conductance of the macroscopic currents was calculated from the peak value of the current through the equation $I=({E}_{m}-{E}_{{rev}})\frac{{G}_{{\max }}}{1+{e}^{({V}_{a}-{E}_{m}/{k}_{a})}}$, where E_rev_ is the estimated reversal potential of the current, and then normalized using the maximum conductance (*G*_*max*_). The activation-voltage relationship was adjusted with a Boltzmann function $G=\frac{{G}_{{\max }}}{1+{e}^{({E}_{m}-{V}_{a}/{k}_{a})}}$, to determine the half-activation potential (*V*_*a*_) and the slope factor (*k*_*a*_). The voltage dependence for inactivation of the L-type calcium channel (LCC) current was evaluated after a pre-pulse of 500 ms for several potentials followed by pulse of 300 ms at 0 mV. The current was plotted against the pre-pulse voltage and the points are presented in mean ± 95% confidence interval to report the precision of each curve. The steady-state curves were fitted with a Boltzmann distribution: $I/{I}_{{\max }}=\frac{1}{1+{e}^{({E}_{m}-{V}_{h}/{k}_{h})}}$, to determine the potential for half the maximum inactivation (V_h_) and slope factor (k_h_). The separation of the K^+^ current components was performed according to Xu *et al*., 1999^13^. The decay of currents recorded at +60 mV potential were fit using the sum of three exponentials: ${(A}_{1}{e}^{-t/{\tau }1})+{(A}_{2}{e}^{-t/{\tau }2})+{(A}_{3}{e}^{-t/{\tau }3})+C$. According to the time constant for inactivation the currents were divided into 3 components *I*_*tof*_, *I*_*tos*_, and *I*_*Ks*_. *A*_*n*_ and *τ*_*n*_ represent each current’s amplitude and time constant, respectively, and *C* corresponds to the sustained component (*I*_*ss*_) of the K^+^ current.

The computational model of cardiac excitation-contraction coupling (ECC) used here is based on our previously published work^14,15^. In short, this local control, compartmental model of ECC consists of ordinary differential equations (ODEs) to capture the time-evolution of various intracellular ion concentrations as well as the deterministic channel gating variables. These ODEs are then coupled to discrete-state, continuous-time Markov chains representing the stochastic gating of the Ca^2+^ channels located within each cardiac Ca^2+^ release unit (CRU). Each CRU in the model consists of 6 sarcolemmal LCCs and 42 of the intracellular, SR Ca^2+^ release channels (ryanodine receptors, RyR2s). The model cell contains 20,000 CRUs and each CRU has individual compartments for its local diadic subspace and junctional SR Ca^2+^ concentrations. These local compartments are then coupled via the bulk cytosolic and network SR [Ca^2+^]. Finally, Hodgkin-Huxley style gating variables are used to generate the various sarcolemmal membrane currents associated with the mouse, cardiac AP. Force and sarcomere length were calculated based on a previously published Markov chain formulation^16^. ECC gain is calculated by dividing the integrated RyR2 flux by the integrated LCC flux. See our previous publication for more details^14,17^. For ARDKO simulations, parameters were modified according to Table S9.

## Results

Unless otherwise noted, experiments were performed using 7 month old mice, when cardiovascular dysfunction has been reported^6,18,19^. Similar to these previous findings, we observed that ARDKO animals displayed increased heart rate (HR) and mean arterial pressure (MAP) secondary to the chronic sympathetic hyperactivity (see Table 1). Further, despite the similar body weight and heart/body weight ratio to the WT group, ARDKO mice presented a slight increase (13%) in VCM membrane capacitance (see Table 1).

**Table 1:** General characteristics of ARDKO animal model.

	Wild Type (WT)	α2a/ α2c KO (ARDKO)
Body weight (g)	31.6 ± 2.4	30.4 ± 1.8 (n = 15 mice)
Heart/Body weight (mg/g)	7.4 ± 0.8	7.7 ± 0.4 (n = 15 mice)
Mean Arterial Pressure (mmHg)	124.0 ± 13.1	137.0 ± 12.1* (n = 15 mice)
Heart Rate (bpm)	540.4 ± 90.7	718.5 ± 23.6* (n = 15 mice)
Cell capacitance (pF)	189.8 ± 35.6 (n = 80 cells)	214.8 ± 56.0* (n = 131 cells)

*P < 0.05; compared to WT (Student’s t-test).

### ARDKO hearts display impaired contractile dynamics

**Figure 1:**
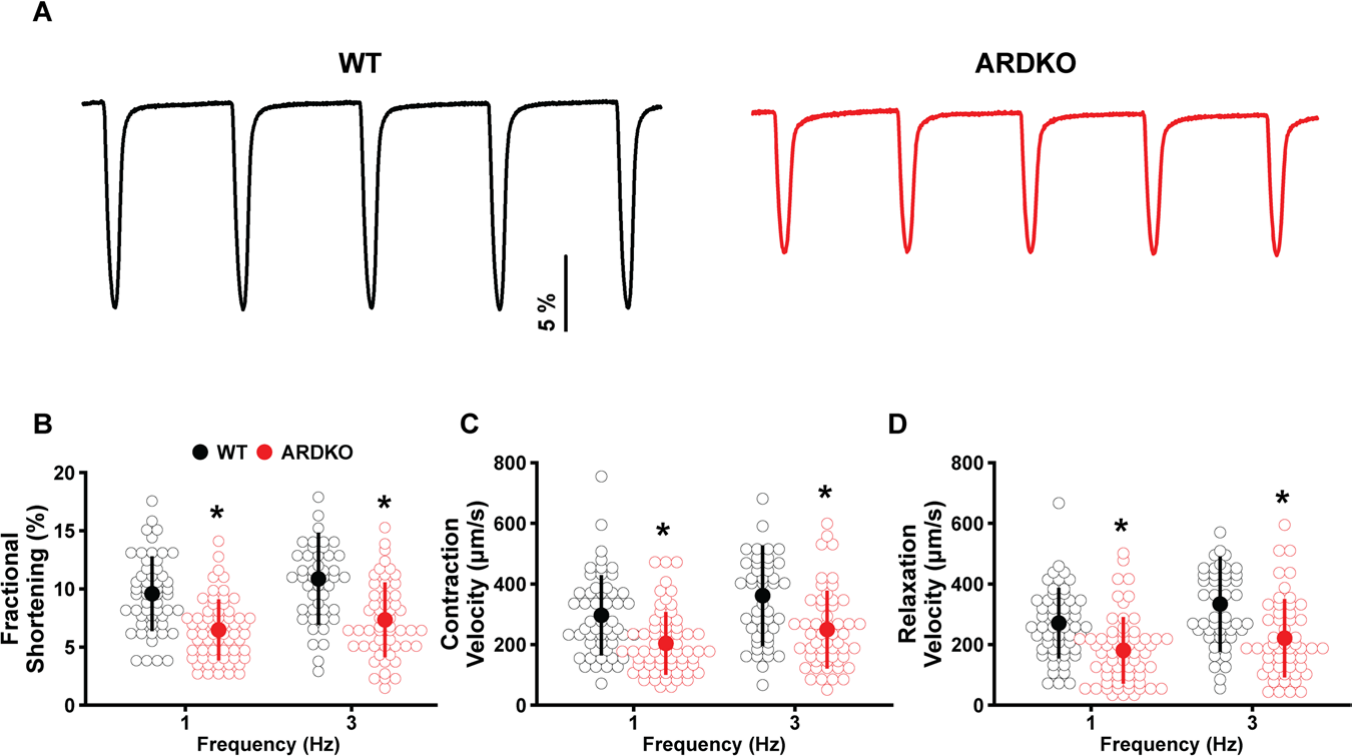
Chronic sympathetic hyperactivity reduces cellular contractility in ventricular cardiomyocytes. Panel (A) shows 5 representative contractions from WT (black) and ARDKO (red) cardiac cells. ARDKO myocytes had a significant reduction in fractional shortening (**B**), contraction (**C**) and relaxation (**D**) velocity. *p < 0.05 when compared to WT at same frequency. All cell contractility data are summarized in Table S5.

Here, we observed that VCMs isolated from ARDKO hearts presented a contractile deficit when compared to WT cells. They also displayed a reduction in the fractional shortening, contraction, and relaxation velocity and a slower relaxation (time to 50% recovery) in both stimulation frequencies (1 and 3 Hz) tested (see Fig. 1 and Table S5) compared to WT. Surprisingly, when VCMs from both groups were exposed to isoproterenol (ISO, 100 nM), the previously observed differences between the WT and ARDKO were abolished (see Table S5). This suggests that although the VCMs have reduced cellular contractility under normal conditions, their adrenergic signaling and cardiac reserve remain intact.

### LCC current is reduced in ARDKO animals

**Figure 2:**
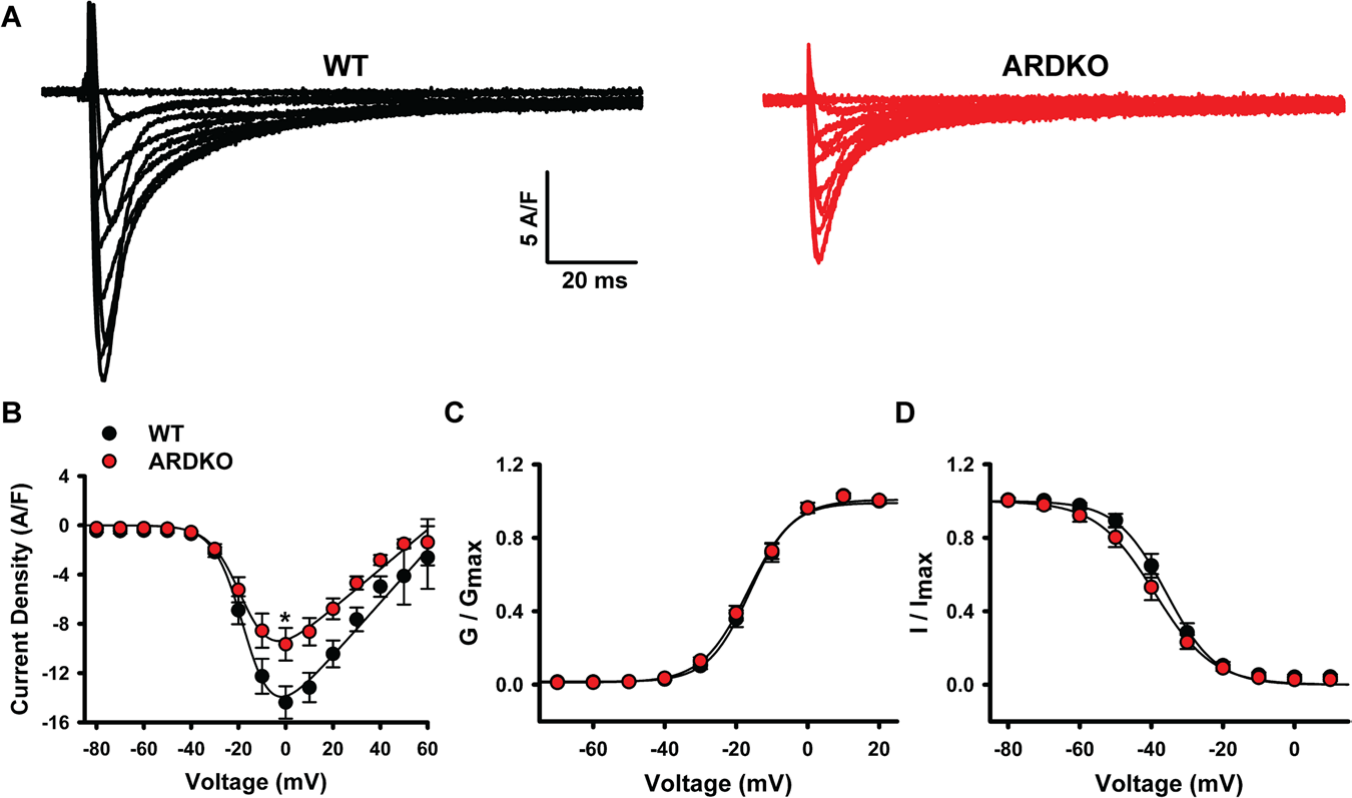
Reduced LCC currents contribute to impaired cellular contractility in ARDKO VCMs. Panel (A) shows representative LCC currents records from WT (black) and ARDKO (red) VCMs. ARDKO had reduced LCC current (**B**), with no significant modulation of stationary voltage-dependency for activation (**C**) and inactivation (**D**) of the LCC. (WT n = 25 cells/4 hearts; ARDKO n = 29 cells/4 hearts). *p < 0.05 when compared to WT.

To better understand the observed reduction in cellular contractility, we evaluated LCC currents, a key component of cardiac ECC. As shown in Fig. 2, the ARDKO VCMs had a lower current density compared to WT cells. Despite the reduced density, no change was observed in the voltage-dependence of LCC activation and inactivation (Fig. 2C). Consistent with the observed changes in cell contractility noted above, when VCMs were stimulated with ISO (100 nM) a similar density of LCC current was observed in both groups (Fig. S1).

### CSH reduces [Ca^2+^]i transients and causes CICR asynchrony

**Figure 3:**
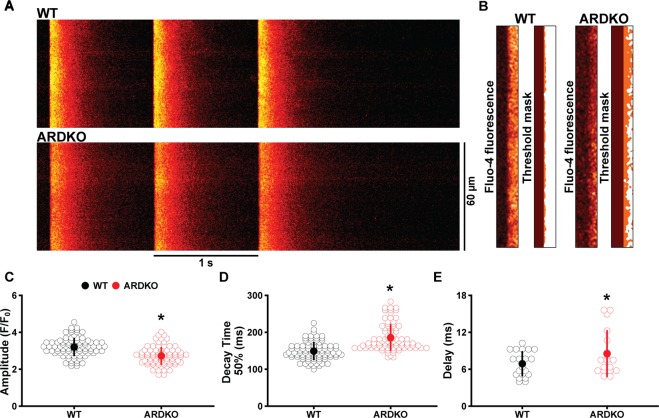
CSH impairs CICR in heart. (Panel **A**) shows confocal line-scan images from WT (Top) and ARDKO (Bottom) VCMs loaded with Fluo-4 Ca^2+^ indicator and paced at 1 Hz. (Panel B) show the initiation phase of [Ca^2+^]_i_ transient used to analyze CICR delay. We used a custom-made routine to create a mask of Fluo-4 fluorescence signals where the orange pixels in the masked image represent the delay between the stimulus and the initiation of CICR. ARDKO VCMs had reduced [Ca^2+^]_i_ transient amplitude (**C**) with slower fluorescence decay (**D**) (WT n = 90 cells/5 hearts; ARDKO n = 83 cells/5 hearts). Also, ARDKO VCMs had delayed initiation of Ca^2+^ release (**E**) (WT n = 20 cells; ARDKO n = 16 cells). *p < 0.05 when compared to WT (Student’s t-test).

In order to further elucidate the influence of CSH on Ca^2+^-induced Ca^2+^ release (CICR) we used confocal microscopy to record [Ca^2+^]_i_ dynamics in quiescent and electrically stimulated cells. [Ca^2+^]_i_ waves were observed in only 8.7% and 11.4% of WT and ARDKO cells, respectively. Within this group, ARDKO appeared to have a higher [Ca^2+^]_i_ wave frequency (0.28 ± 0.10 waves 10 s^−1^) over their WT counterparts (0.12 ± 0.05 waves 10 s^−1^), however this was not statistically different between groups. When electrically stimulated (1 Hz), ARDKO VCMs had reduced [Ca^2+^]_i_ transient amplitudes when compared to their WT counterparts (Fig. 3A). Additionally, the [Ca^2+^]_i_ transient of the ARDKO VCMs had a slower decay when compared to WT, but rise time was unchanged (Fig. 3C,D). While the rise times of the [Ca^2+^]_i_ transients were similar, the synchrony of CICR was altered in ARDKO. In fact, ARDKO VCMs displayed a significant delay in initiation of Ca^2+^ release (Fig. 3B,E), which likely underlies the reduced contraction velocity.

### Cardiac AP is prolonged following CSH

**Figure 4:**
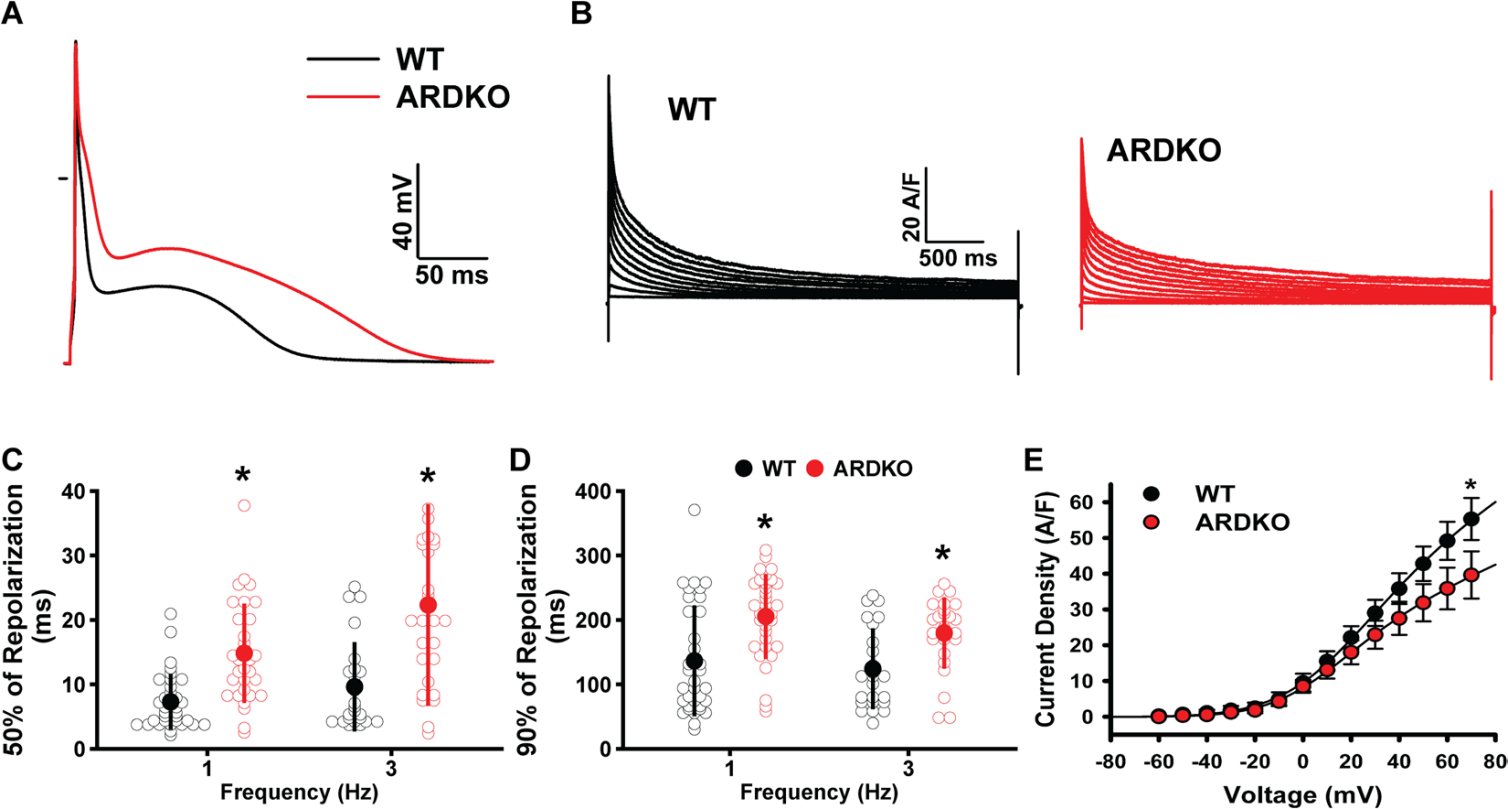
ARDKO displayed AP prolongation and reduced voltage-gated outward K^+^ currents. Panel A and B are representative traces of an action potential (**A**) and outward K^+^ currents (**B**) recorded from WT (black) and ARDKO (red) myocytes. ARDKO VCMs displayed delayed AP repolarization, as demonstrated by the increase in time to reach 50% (**C**) and 90% (**D**) of repolarization (WT n = 34 cells; ARDKO n = 32 cells). The reduced voltage-gated outward K^+^ currents (**E**) contribute to the prolonged AP waveform profile in ARDKO (WT n = 31 cells/4 hearts; ARDKO n = 34 cells/4 hearts). *p < 0.05 when compared to WT.

To gain a better understanding of how CSH modulates cardiac ECC dynamics, we recorded action potentials (APs) of WT and ARDKO VCMs using a whole-cell patch-clamp technique. A marked prolongation of the AP was observed in ARDKO (Fig. 4A). Specifically, there was an increase in repolarization times (i.e., time to 50% and 90% repolarization) in the ARDKO group when compared to WT. Despite drastic changes in repolarization, the other AP parameters related to depolarization (i.e., amplitude, maximal depolarization rate, and resting potential) were similar in both groups. All values obtained in the AP experiments of the 7-month-old animals are contained in Table S6.

*CSH is associated with reduced outward K*^*+*^ *currents and increased NCX current*.

In VCMs of 7-month-old ARDKO animals we observed reduced cellular contractility, delayed relaxation, and AP prolongation when compared to WT. To characterize the cellular basis that underlies this delayed repolarization, we conducted experiments to identify the specific ion currents altered in ARDKO. Specifically, the outward and inward K^+^ currents as well as the NCX current. Similar to the LCC current, outward K^+^ current density is reduced in the ARDKO cells when compared to WT (Fig. 4). The decay of the outward currents generated at 60 mV was fit using a sum of three exponentials (see in Materials and Methods) as a way to discriminate the contribution of K^+^ current subtypes. This analysis indicated that the K^+^ current most altered in the ARDKO VCMs was the fast component of the transient outward current (*I*_*tof*_) (Fig. S2). Although the density of other K^+^ currents remains similar between the groups, due to the reduction of *I*_*tof*_, the slow (*I*_*Ks*_) and sustained (*I*_*ss*_) currents have a larger contribution to the sum of the outward K^+^ currents in ARDKO. The inward rectifier currents are key elements during later phases of AP repolarization and also help maintain adequate resting membrane potential. Consistent with the unchanged membrane resting potential of ARDKO, no significant change in those currents could be detected (Fig. S3).

**Figure 5:**
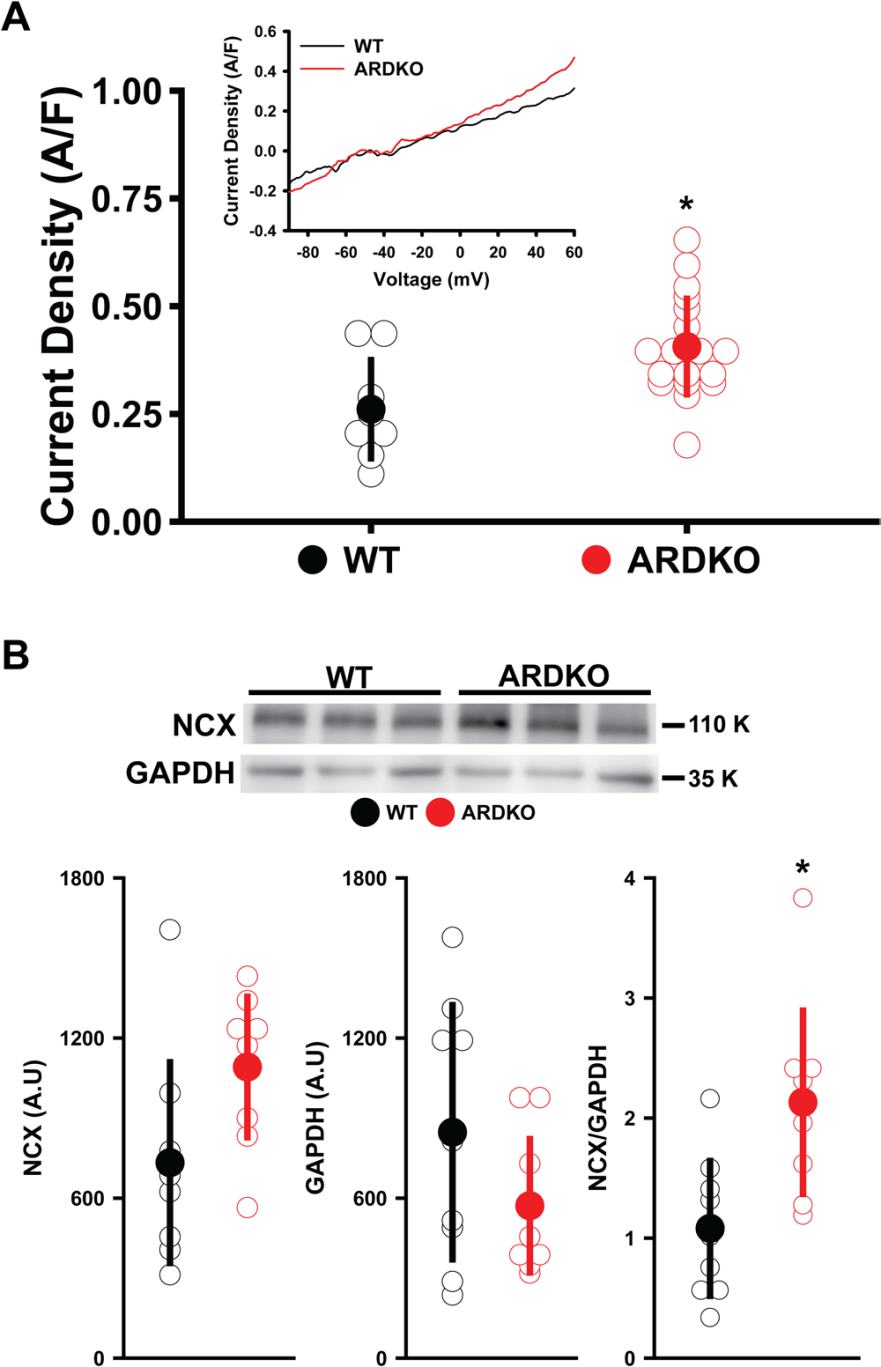
CSH increases NCX expression and current density. ARDKO VCMs have a significant increase in NCX current density recorded in whole-cell patch clamp (**A**) (WT n = 18 cells/3 hearts; ARDKO n = 9 cells/3 hearts). The inset in panel A shows representative traces NCX currents from WT (black) and ARDKO (red) myocytes. NCX currents reduction associated with an overexpression of the NCX protein (**B**) (n = 9 samples for each group). *p < 0.05 when compared to WT (Mann-Whitney). Inset of panel B shows bands cropped from the same blot for each respective protein. The full-length blots can be found in Figure S5.

In addition to participating in the Ca^2+^ extrusion from the cytosol, NCX is an electrogenic transporter and can therefore influence the transmembrane potential. This is most commonly observed during the repolarization phase of the cardiac AP. ARDKO VCMs had an increase (55%) in the current sensitive to 5 mM of Ni^2+^ (a blocker of NCX activity) over WT (see Fig. 5A). In accordance with these findings, VCMs from the ARDKO animals also displays a higher expression of NCX protein levels (Fig. 5B). Importantly, consistent evidence points to oxidative stress as an important modulator of the cardiac ECC. Here we observed the modulation of electromechanical properties of ARDKO VCMs was accompanied by an increase in reactive oxygen species (ROS) production but no significant change in nitric oxide levels (see Fig. S4).

### Brief periods of CSH induced no significant changes in the electromechanical properties of VCMs

In order to determine if the altered ECC in ARDKO arose as an adaptation to chronic sympathetic hyperactivity, we repeated experiments in younger (i.e., 3 months old versus 7 months old) animals. While 3-month-old ARDKO animals displayed reduced body weight compared to the WT, there was no alteration in their heart/body weight ratio to indicate heart pathology. Consistent with this, we observed that 3 month old ARDKO VCMs presented similar cellular contractility (compared to WT) in both stimulation paradigms and upon activation of the βAR (Table S7). Additionally, APs recorded from both groups were similar across all analyzed parameters. This supports our hypothesis that sympathetic hyperactivity must be sustained for long periods to induce maladaptation on cardiac ECC. The cellular contractility and AP parameters from 3-month-old animals are summarized in Tables S7 and S8, respectively.

### Computational modeling reveals molecular basis for abnormal calcium handling driven by CSH

**Figure 6:**
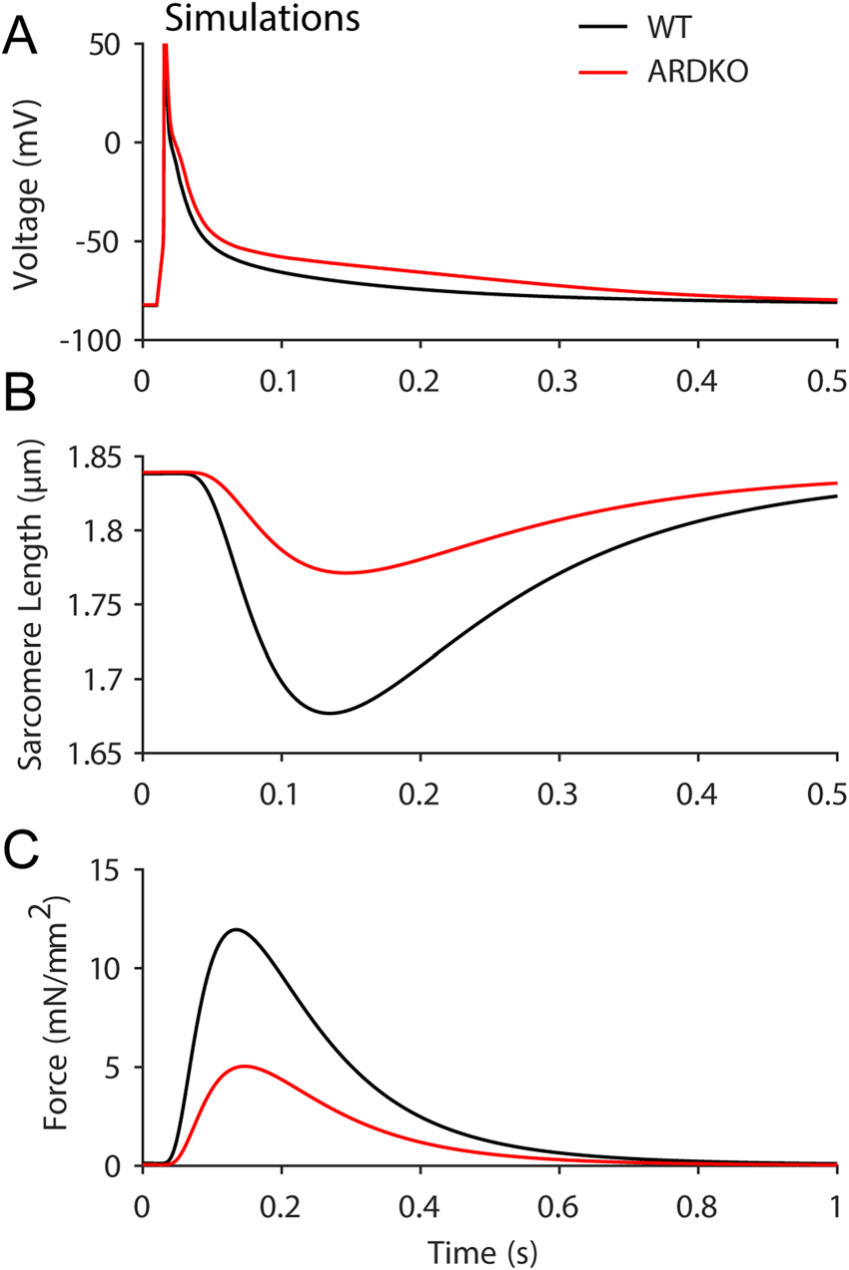
Simulations of cardiac CICR suggests impaired ECC in ARDKO mice. (**A**) Field-stimulated AP for WT (black) and ARDKO (red) simulations. (**B**) Sarcomere shortening for WT (black) and ARDKO (red) during APs shown in (A). (**C**) Force generated during the simulated APs.

We utilized our established computational model of cardiac CICR and ECC and constrained key parameters with the quantified experimental measures from the ARDKO cardiomyocytes (i.e., reduced voltage-gated K^+^ and Ca^2+^ currents along with increased NCX current) to create a representation of WT and ARDKO conditions. Importantly, we observed that altering only these key parameters account for the AP prolongation and reduced force generation in ARDKO (see Fig. 6).

**Figure 7:**
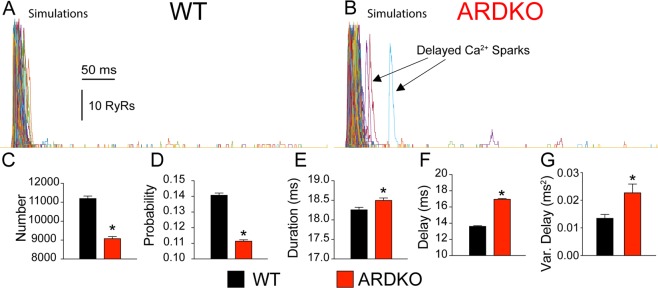
Computational modeling provides mechanistic insights into how CSH alters Ca^2+^ spark properties. Panel A&B) Simulated Ca^2+^ sparks (shown as number of open RyR2 channels) during the APs shown in Fig. 6. in WT (**A**) and ARDKO (**B**) where each colored line indicates RyR2 cluster activity from a different CRU. *For clarity only 10% of events are shown*. (**C**) Average number of Ca^2+^ sparks triggered during a [Ca^2+^]_i_ transient. (**D**) Probability of an LCC opening triggering a Ca^2+^ spark. **E**) Average Ca^2+^ spark duration. Ca^2+^ spark synchrony measured as average delay time from start of the AP to each Ca^2+^ spark peak (**F**) and variance of delay (**G**). In panels C-G, WT is indicated by black bars and ARDKO by red bars. (n = 10 simulations for each group) *p < 0.05 when compared to WT (Mann-Whitney).

Additionally, the model details the Ca^2+^ spark behavior in the ARDKO myocytes (Fig. 7). CSH decreased the total number of Ca^2+^ sparks during an AP (Fig. 7C) which led to a reduced [Ca^2+^]_i_ transient and force production in the ARDKO VCMs. This was directly correlated with impairment of LCC current observed in ARDKO myocytes, causing the probability of an LCC opening triggering a Ca^2+^ spark (Fig. 7D) to be reduced. This also led to a significant reduction in CICR synchrony, observed in the simulation by delayed Ca^2+^ sparks (Fig. 7B,F) and increased variability of Ca^2+^ spark timing (Fig. 7G). This evidence, along with the experimental data presented above, suggests that impaired CICR contributes to the altered heart function observed in the ARDKO heart that underlies the progression to cardiac dysfunction.

**Figure 8:**
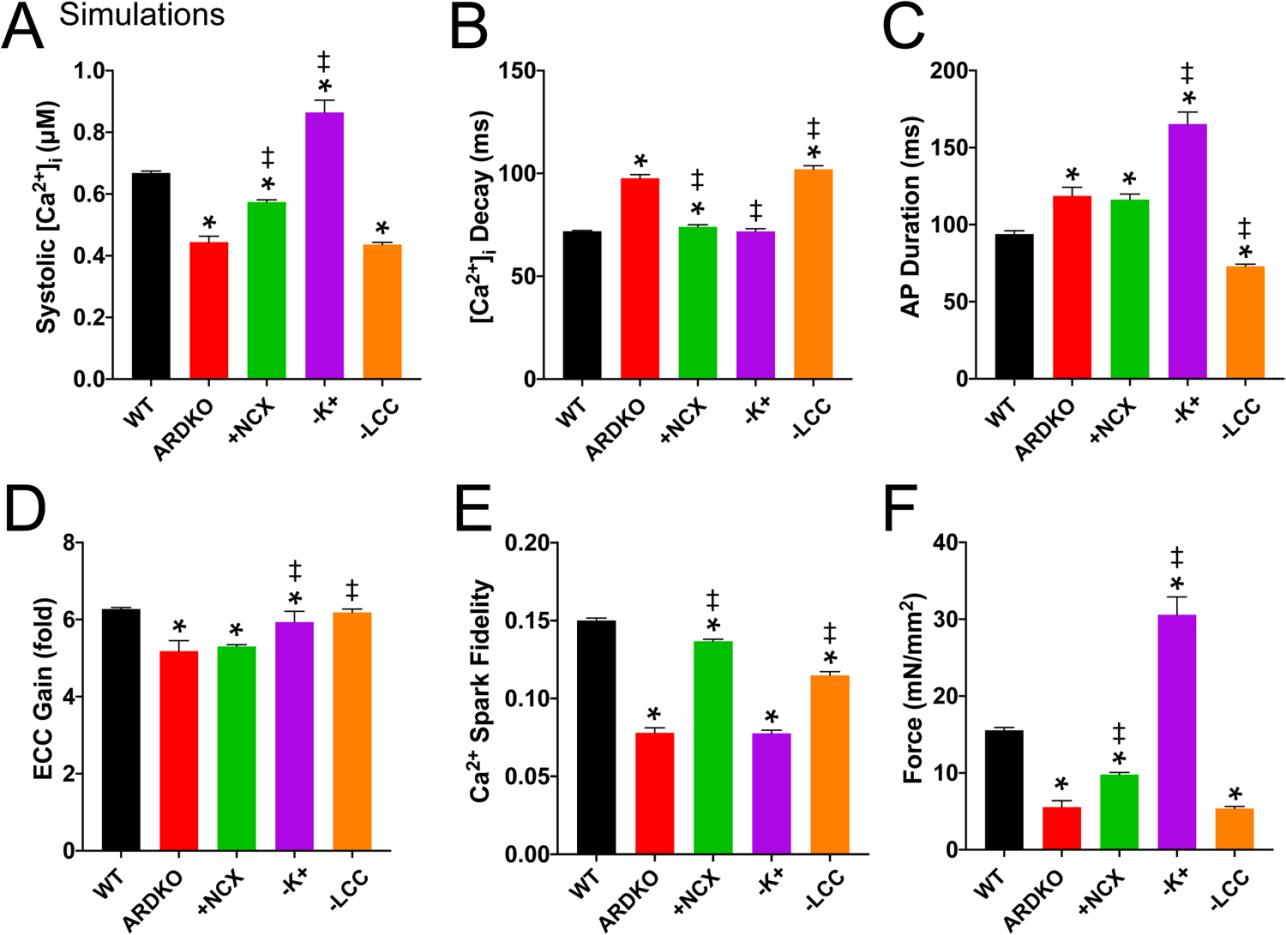
Simulations reveal individual contributions of membrane current changes on ARDKO phenotype. Peak systolic [Ca^2+^]_i_ transient **(A)**, Decay time constant for [Ca^2+^]_i_ transient **(B)**, time to 90% AP recovery **(C)**, EC coupling gain (**D)**, Ca^2+^ spark fidelity to LCC openings **(E)** and contractile force **(F)** for WT (black bars), ARDKO (red bars), and conditions where each membrane current change associated with ARDKO is implemented in isolation (green, purple, and orange bars for NCX, I_K_^+^, and LCC, respectively. Significance (p < 0.05, One-way ANOVA) measured against WT (*) and against ARDKO (‡) and n = 10 simulation runs for each condition.

To better understand the individual contribution of the experimentally observed, membrane current changes (i.e., LCC, NCX, and I_K_^+^) to the ARDKO phenotype, we performed simulations where each current alteration was implemented individual. These simulations demonstrate that: (1) the reduced LCC current primarily drives changes in Ca^2+^ transient (i.e., reduced amplitude and slower decay) (see Fig. 8A,B) which in turn produces reduced force (see Fig. 8F). Note, that reduced LCC current shortens the AP (as expected) possibly blunting the severity of AP prolongation in ARDKO (Fig. 8C).; (2) elevated NCX current contributes to reduced Ca^2+^ transient amplitude and AP prolongation but perhaps most significantly drives decreased EC coupling gain (Fig. 8D)^20^; and finally (3) diminished I_K_^+^ currents drive elevated [Ca^2+^]_i_ transient amplitude and AP prolongation (as expected) but also increase force production (Fig. 8F). Interestingly, changes to I_K_^+^ currents in ARDKO significantly alter “Ca^2+^ spark fidelity” from WT. “Ca^2+^ spark fidelity” is a term used to describe the likelihood of single channel opening (in this case, an LCC) within a CRU yielding the activation of the CRU RyR2 cluster to generate a Ca^2+^ spark (see Williams *et al*., Biophys J., 2011 and Wescott *et al*., J Mol Cell Cardiol., 2016 for more information regarding the implications of altered Ca^2+^ spark fidelity^14,15^). Furthermore, the diminished Ca^2+^ spark fidelity observed in the ARDKO compared to WT (see Fig. 8E) suggests that diminished systolic [Ca^2+^]_i_ transient and contractile dysfunction that follows is due to diminished coupling between the LCC and RyR2 and not altered SR Ca^2+^ content. While SR Ca^2+^ content was not measured experimentally, the model predicted similar diastolic SR [Ca^2+^] (~630 μM) but slightly altered systolic levels for WT and ARDKO (~480 and ~530 μM, respectively).

## Discussion

In this study we demonstrated that the genetic model of CSH provided by the ARDKO mouse induces contractile dysfunction in VCMs by remodeling of various ion channels and transporters essential for the ECC. These changes were observed before a severe hypertrophy and without compromised adrenergic signaling.

### ARDKO Mouse model of sympathetic hyperactivity

We used a genetic model of sympathetic hyperactivity caused by the knock-out of α2A and α2C adrenergic receptors. This double knock-out leads to sympathetic hyperactivity and increased plasmatic norepinephrine, and in the long term these animals are prone to developing cardiac hypertrophy, reduced physical capacity, impaired cardiac contractility, altered VCM ultrastructure, and higher mortality with age progression^6,7^. The reduced contractility displayed by ARDKO hearts is a common point on various cardiac disorders including HF, especially at higher heart rates or in the presence of catecholamines^21^. In this study, the model of endogenous sympathetic overactivity generated by deletion of adrenergic α2A and α2C receptors reduced fractional shortening and contraction and relaxation velocity (see above) which correlates well with past observations^6^.

The cardiac dysfunction in this animal model can be attenuated by β-AR antagonists treatment (i.e., Metoprolol and Carvedilol)^8^, which is consistent with the proposed role of these receptors in cardiac maladaptation in ARDKO model. Furthermore, the cardiac ECC remodeling in the ARDKO, is also consistent with other experimental models in which prolonged β-AR stimulation by isoproterenol resulted in reduced cardiac contractility^22–25^. While cardiac ultrastructure, capacity, and contractility have already been extensively characterized, here we provided specific evidence for ECC alterations and identified the underlying mechanisms responsible for the macroscopic changes previously reported in this experimental model^6^.

One characteristic of the HF pathology is the abnormal β-AR signaling via desensitization of those receptors^26,27^. In the heart, this desensitization is regulated by G-protein-coupled receptor kinase 2 (GRK2), which is elevated in HF^26,28^. While, the upregulation of GRK2 in the adrenal medulla also play a critical role in the elevated circulating levels of catecholamines associated to HF^27,29,30^. However, at the time points included in our study (3 and 7 months), CSH had not impaired β-AR signaling. When exposed to isoproterenol, cardiomyocytes from WT and ARDKO had similar contractile characteristics and LCC current density, in contrast to the reduction observed under normal conditions. Furthermore, prior *in vivo* observations, showed no difference in the maximal chronotropic response between WT and ARDKO mice when challenged with isoproterenol^6^. Since GRK2 and β-AR expression levels are unknown in ARDKO mice, the molecular background for β-AR signaling is not clear in this CSH model. In the future, the availability of such data combined with the results shown here will better characterize the role of CSH in driving cardiac remodeling. Taken together this suggests that CSH can compromise cardiac ECC prior to β-AR desensitization. However, we do not exclude the possibility that CSH can impair β-AR signaling if sustained for longer periods than those explored in this study.

### CSH Drives electrical remodeling of the sarcolemma

Another important change found in animals with CSH was an increase in AP duration. This is consistent with early stage HF studies where AP prolongation is commonly observed and is believed to be an important cellular mechanism for maintaining force generation as a compensatory mechanism after the contractile dysfunction associated with HF^31^. AP prolongation, however, fails to maintain force generation at later stages of HF and becomes a driving factor for the onset of arrhythmic events^32,33^. Here, we show that CSH leads to a similar AP prolongation and that reduced outward K^+^ currents and elevated NCX activity underlie this profile. In parallel, sarcolemmal LCC current density is reduced in the ARDKO model with significant implications for CICR dynamics and cell contraction.

The reduced K^+^ currents associated with CSH are also observed in other models of HF^24,32,34^. Following a detailed analysis of the contribution of the various K^+^ current subtypes, we found no changes in delayed rectifier and inward rectifier currents. In larger mammals, these subtypes are important regulators of AP duration, and in late stage HF, decreased inward rectifier currents play an important role in the generation of arrhythmias^35^. However, delayed rectifier K^+^ currents have low expression in small rodents and play no significant role in the AP prolongation observed in the CSH-induced model. In fact, the major contribution to down-regulation of outward K^+^ currents in our experiments was a reduction in *I*_*tof*_. The reduction in *I*_*tof*_ is another common maladaptation related to K^+^ channels in HF. Note, however, that while this current significantly regulates AP duration in small rodents it plays a minor role in larger mammals despite being responsible for the initial repolarization phase (phase 1) of the cardiac AP^36,37^. Phase 1 governs the initial Ca^2+^ influx through LCC and therefore CICR and contractility. Hence, a more prominent phase 1 repolarization due to a higher density of *I*_*tof*_ would likely increase Ca^2+^ influx (due to elevated driving force for Ca^2+^ entry) thereby triggering more Ca^2+^ release from the SR and consequently improving myocyte contraction^38^. Conversely, down-regulation of *I*_*tof*_ would likely contribute to the reduced contractility observed during HF and in the ARDKO model.

Increased NCX expression and its role in controlling intracellular Ca^2+^ levels have been previously described in the ARDKO experimental model^18^. In this study, we provide new evidence associating increased NCX current with the prolongation of the AP in the ARDKO. This increase in NCX levels might represent a compensatory mechanism for the decreased SERCA function highlighted by slowed decay of Ca^2+^ transients in ARDKO. Such a mechanism would act as a means for the cell to attempt to maintain normal relaxation rates^39^. Note that in ARDKO, reduced SERCA levels have also been shown to be partly compensated for by increased phospholamban phosphorylation (in Thr17 but not Ser16)^18,40^. The increase in NCX levels may also play an important role in HF when in reverse mode, which can aid in Ca^2+^ entry during the systolic phase to augment the pathologically diminished SR Ca^2+^ release^39,41^. Finally, NCX transport is electrogenic and therefore NCX activity can alter the cardiac AP. This is especially true in HF and in larger animals, where NCX current can drastically alter the shape of the AP and drive arrhythmias^42,43^.

Consistent with a prominent role of NCX is evidence in healthy mice that an inducible overexpression of NCX1 causes a pronounced prolongation of the AP without significantly changing the resting potential^44^. Furthermore, an increase in ROS availability, as identified here, has been linked to an reduced I_tof_ current density^45,46^ and increased activity of NCX^41^ which would promote the AP prolongation observed in the ARDKO myocytes. Furthermore, an unbalanced cellular redox state reduces SERCA function and increases the SR Ca^2+^ leakage via RyR2, which can lead to Ca^2+^ mishandling and arrhythmias^47,48^.

In addition to the reduced fractional shortening and contraction and relaxation velocity displayed by the endogenous sympathetic hyperactivity model used here, a reduction in LCC current density was also observed. Some isoproterenol-based sympathetic hyperactivity models show decreased Ca^2+^ currents^24,49^, however, others observe a maintenance of the macroscopic current^22^. This mismatch may be due to a non-uniformity of isoproterenol administration, where some results vary according to the animal model, dose, and time of treatment. The reduced Ca^2+^ currents induces a directly contractile dysfunction process^50^. However, in HF, reduced cell contractility could not be well correlated with decreased LCC current density^39^ which appears more frequently in end-stages of HF^32^. In addition, decreased expression of LCC was not observed in all cases^51^. However, there is a considerable redistribution, with a decrease in Ca^2+^ current in the t-tubule membrane, likely due to t-tubule remodeling, with a concomitant increase in sarcoplasmic membrane surface area^22^. This t-tubule remodeling is an important factor in the transition between a hypertrophic state and HF, as it has close relationship with contractile function^52^.

Here, in the ARDKO heart we observed an approximately two-fold reduction in peak LCC current density (see above). The two likely mechanisms that contribute to this reduced LCC current are: 1) cellular hypertrophy, and the resulting increase in cell capacitance, found in the ARDKO model and other models involving the exogenous β-adrenergic stimulation (e.g., Isoproterenol)^22,49^. 2) increased expression or activity of phosphatases (i.e., protein phosphatase 1, PP1) in the ARDKO heart that are known to reduce LCC phosphorylation levels and LCC currents^18,19^. Additionally, the expression of some kinases important for the modulation of LCC (e.g., CaMKII) are not altered in the animal model used in this study^19^. Given the critical nature of LCC in initiating CICR during systole to generate the [Ca^2+^]_i_ transient, we decided to investigate how this reduced LCC current would alter CICR dynamics in the ARDKO heart.

### Dyssynchronous Ca release contributes to contractile dysfunction in ARDKO hearts

Consistent with past studies we observed a significant decrease in contractile function in the ARDKO hearts. While commonly observed via reduced ejection fraction^6^, this deficit was highlighted here via a significant decrease in single cell fractional shortening from freshly-isolated, field-stimulated, VCMs. While the marked decrease in LCC currents described above is a likely contributor to this reduced shortening we investigated further using confocal recording of [Ca^2+^]_i_ transients.

Reduced LCC current in ARDKO hearts impairs CICR therefore causing reduced [Ca^2+^]_i_ transient amplitude and asynchrony of initial Ca^2+^ release. Using a computational model of cardiac ECC, we observed increased variability in timing of Ca^2+^ sparks leading to a significant reduction in Ca^2+^ release synchrony and higher incidence of delayed Ca^2+^ sparks during the [Ca^2+^]_i_ transient in ARDKO heart, which contributed to slowed [Ca^2+^]_i_ transient decay. Also, by isolating individual currents, the simulations revealed some complex interactions between the various components of ARDKO-based remodeling. This was especially notable when relating to the opposing influences of the reduced I_K_^+^ and LCC currents on the systolic Ca^2+^ transient and force generation. Since this Ca^2+^ mishandling and impaired force generation underlies contractile dysfunction^39^, our experimental and computational data combine to better characterize the role of CSH in cardiac dysfunction and disease progression.

### Summary

Chronic sympathetic hyperactivity (CSH) induced by deletion of the α2A and α2C adrenergic receptors reduces contractility in VCMs by an impairment of Ca^2+^ signaling. These changes are accompanied by a prolongation of the cardiac action potential caused by reduced outward K^+^ currents and increased NCX current. These changes along with diminished LCC current drive dysfunctional CICR behavior at the molecular level which underlies the macroscopic contractile deficits. These results have provided novel insights into the role of CSH in the progression of cardiovascular disorders.

## Supplementary information

Supplementary Information.

## References

[1] Triposkiadis F (2009). The sympathetic nervous system in heart failure physiology, pathophysiology, and clinical implications. J. Am. Coll. Cardiol..

[2] Charkoudian N, Rabbitts JA (2009). Sympathetic neural mechanisms in human cardiovascular health and disease. Mayo Clin. Proc..

[3] Lymperopoulos A, Rengo G, Koch WJ (2013). Adrenergic nervous system in heart failure: pathophysiology and therapy. Circ. Res..

[4] Mann DL, Bristow MR (2005). Mechanisms and models in heart failure: the biomechanical model and beyond. Circulation.

[5] Floras JS (2009). Sympathetic nervous system activation in human heart failure: clinical implications of an updated model. J. Am. Coll. Cardiol..

[6] Brum PC, Kosek J, Patterson A, Bernstein D, Kobilka B (2002). Abnormal cardiac function associated with sympathetic nervous system hyperactivity in mice. Am. J. Physiol. Hear. Circ. Physiol.

[7] Hein L, Altman JD, Kobilka BK (1999). Two functionally distinct alpha2-adrenergic receptors regulate sympathetic neurotransmission. Nature.

[8] Bartholomeu JB (2008). Intracellular mechanisms of specific beta-adrenoceptor antagonists involved in improved cardiac function and survival in a genetic model of heart failure. J. Mol. Cell Cardiol..

[9] Shioya T (2007). A simple technique for isolating healthy heart cells from mouse models. J. Physiol. Sci..

[10] Barman P, Choisy SCM, Hancox JC, James AF (2011). β-Adrenoceptor/PKA-stimulation, Na+−Ca2+ exchange and PKA-activated Cl- currents in rabbit cardiomyocytes: A conundrum. Cell Calcium.

[11] Kalyanaraman B (2012). Measuring reactive oxygen and nitrogen species with fluorescent probes: Challenges and limitations. Free. Radic. Biol. Med..

[12] Lowry OH, Rosebrough NJ, Farr AL, Randall RJ (1951). Protein measurement with the Folin phenol reagent. J. Biol. Chem..

[13] Xu H, Guo W, Nerbonne JM (1999). Four kinetically distinct depolarization-activated K+ currents in adult mouse ventricular myocytes. J. Gen. Physiol..

[14] Wescott AP, Jafri MS, Lederer WJ, Williams GSB (2016). Ryanodine receptor sensitivity governs the stability and synchrony of local calcium release during cardiac excitation-contraction coupling. J. Mol. Cell. Cardiol..

[15] Williams GSB (2011). Dynamics of calcium sparks and calcium leak in the heart. Biophys. J..

[16] Mullins PD, Bondarenko VE (2013). A Mathematical Model of the Mouse Ventricular Myocyte Contraction. PLoS One.

[17] Joca, H. C., Coleman, A. K., Ward, C. W. & Williams, G. S. B. Quantitative tests reveal that microtubules tune the healthy heart but underlie arrhythmias in pathology. *J. Physiol*., 10.1113/JP277083 (2019). 10.1113/JP277083 PMC7432954 30582750

[18] Rolim NP (2007). Exercise training improves the net balance of cardiac Ca2+ handling protein expression in heart failure. Physiol. Genomics.

[19] Oliveira RS (2009). Cardiac anti-remodelling effect of aerobic training is associated with a reduction in the calcineurin/NFAT signalling pathway in heart failure mice. J. Physiol..

[20] Cheng H (1996). Excitation-contraction coupling in heart: new insights from Ca2+ sparks. Cell Calcium.

[21] Houser SR, Margulies KB (2003). Is depressed myocyte contractility centrally involved in heart failure?. Circ. Res..

[22] Horiuchi-Hirose M (2011). Decrease in the density of t-tubular L-type Ca2+ channel currents in failing ventricular myocytes. Am. J. Physiol. Hear. Circ. Physiol.

[23] Suzuki M (1998). Altered inotropic response of endothelin-1 in cardiomyocytes from rats with isoproterenol-induced cardiomyopathy. Cardiovasc. Res..

[24] Zhang LM, Wang Z, Nattel S (2002). Effects of sustained beta-adrenergic stimulation on ionic currents of cultured adult guinea pig cardiomyocytes. Am. J. Physiol. Hear. Circ. Physiol.

[25] Youn JY (2013). Oxidative stress in atrial fibrillation: an emerging role of NADPH oxidase. J. Mol. Cell Cardiol..

[26] Huang ZM, Gold JI, Koch WJ (2011). G protein-coupled receptor kinases in normal and failing myocardium. Front. Biosci..

[27] Reinkober J (2012). Targeting GRK2 by gene therapy for heart failure: Benefits above β-blockade. Gene Ther..

[28] Choi DJ, Koch WJ, Hunter JJ, Rockman HA (1997). Mechanism of β-adrenergic receptor desensitization in cardiac hypertrophy is increased β-adrenergic receptor kinase. J. Biol. Chem..

[29] Lymperopoulos A, Rengo G, Funakoshi H, Eckhart AD, Koch WJ (2007). Adrenal GRK2 upregulation mediates sympathetic overdrive in heart failure. Nat. Med..

[30] Cannavo A (2017). GRK2 Regulates α2-Adrenergic Receptor–Dependent Catecholamine Release in Human Adrenal Chromaffin Cells. J. Am. Coll. Cardiology.

[31] Bers DM (2002). Cardiac excitation-contraction coupling. Nature.

[32] Aiba T, Tomaselli GF (2010). Electrical remodeling in the failing heart. Curr. Opin. Cardiol..

[33] Coronel R (2013). Electrophysiological changes in heart failure and their implications for arrhythmogenesis. Biochim. Biophys. Acta.

[34] Aflaki M (2014). Exchange protein directly activated by cAMP mediates slow delayed-rectifier current remodeling by sustained beta-adrenergic activation in guinea pig hearts. Circ. Res..

[35] Myles RC, Wang L, Bers DM, Ripplinger CM (2015). Decreased inward rectifying K+ current and increased ryanodine receptor sensitivity synergistically contribute to sustained focal arrhythmia in the intact rabbit heart. J. Physiol..

[36] Greenstein JL, Wu R, Po S, Tomaselli GF, Winslow RL (2000). Role of the calcium-independent transient outward current I(to1) in shaping action potential morphology and duration. Circ. Res..

[37] Sun X, Wang HS (2005). Role of the transient outward current (Ito) in shaping canine ventricular action potential–a dynamic clamp study. J. Physiol..

[38] Dong M (2010). Role of the transient outward current in regulating mechanical properties of canine ventricular myocytes. J. Cardiovasc. Electrophysiol..

[39] Bers DM (2006). Altered cardiac myocyte Ca regulation in heart failure. Physiol..

[40] Medeiros A (2008). Exercise training delays cardiac dysfunction and prevents calcium handling abnormalities in sympathetic hyperactivity-induced heart failure mice. J. Appl. Physiol..

[41] Dipla K, Mattiello JA, Margulies KB, Jeevanandam V, Houser SR (1999). The sarcoplasmic reticulum and the Na+/Ca2+ exchanger both contribute to the Ca2+ transient of failing human ventricular myocytes. Circ. Res..

[42] Armoundas AA, Hobai IA, Tomaselli GF, Winslow RL, O’Rourke B (2003). Role of sodium-calcium exchanger in modulating the action potential of ventricular myocytes from normal and failing hearts. Circ. Res..

[43] Hobai IA, O’Rourke B (2000). Enhanced Ca(2+)-activated Na(+)-Ca(2+) exchange activity in canine pacing-induced heart failure. Circ. Res..

[44] Wang J (2009). Induced overexpression of Na+/Ca2+ exchanger transgene: altered myocyte contractility, [Ca2+]i transients, SR Ca2+ contents, and action potential duration. Am. J. Physiol. Hear. Circ. Physiol.

[45] Rozanski GJ, Xu Z (2002). Sulfhydryl modulation of K+ channels in rat ventricular myocytes. J. Mol. Cell Cardiol..

[46] Drum BM (2016). Oxidative stress decreases microtubule growth and stability in ventricular myocytes. J. Mol. Cell Cardiol..

[47] Zima AV, Copello JA, Blatter LA (2004). Effects of cytosolic NADH/NAD(+) levels on sarcoplasmic reticulum Ca(2+) release in permeabilized rat ventricular myocytes. J. Physiol..

[48] Wagner S, Rokita AG, Anderson ME, Maier LS (2013). Redox regulation of sodium and calcium handling. Antioxid. Redox Signal..

[49] Zhang ZS (2005). Enhanced inhibition of L-type Ca2+ current by beta3-adrenergic stimulation in failing rat heart. J. Pharmacol. Exp. Ther..

[50] Goonasekera SA (2012). Decreased cardiac L-type Ca2+ channel activity induces hypertrophy and heart failure in mice. J. Clin. Invest..

[51] Pitt GS, Dun W, Boyden PA (2006). Remodeled cardiac calcium channels. J. Mol. Cell Cardiol..

[52] Wei S (2010). T-tubule remodeling during transition from hypertrophy to heart failure. Circ. Res..

