# Supplementary material for: Chronic Sympathetic Hyperactivity Triggers Electrophysiological Remodeling and Disrupts Excitation-Contraction Coupling in Heart
Source: Sci Rep. 2020 May 14;10:8001. doi: 10.1038/s41598-020-64949-7 (PMC7224293; doi:10.1038/s41598-020-64949-7)
Supplement: Supplementary file 1 — Supplementary Information. [file 41598_2020_64949_MOESM1_ESM.docx]

**Supplemental Information**

**Chronic Sympathetic Hyperactivity Triggers Electrophysiological Remodeling and Disrupts Excitation-Contraction Coupling in Heart**

Humberto C. Joca^1,2^, Artur Santos‐Miranda^1^, Julliane V. Joviano‐Santos^3^, Rebeca P. M. Maia-Joca^1^, Patricia C. Brum^4^, George S. B. Williams^2^, Jader S. Cruz^1^*

^1^ Department of Biochemistry and Immunology, Institute of Biological Sciences, Federal University of Minas Gerais, Belo Horizonte, MG, Brazil

^2^ Center for Biomedical Engineering and Technology, University of Maryland School of Medicine, Baltimore, Maryland

^3^ Department of Morphology, Institute of Biological Sciences, Federal University of Minas Gerais, Belo Horizonte, MG, Brazil

^4^ School of Physical Education and Sport, University of São Paulo, São Paulo, SP, Brazil

* Corresponding author: Jader S. Cruz - Department of Biochemistry and Immunology, Federal University of Minas Gerais, Av. Antônio Carlos, 6627 – 31.270‐901 – Belo Horizonte, MG, Brazil. Email: jcruz@icb.ufmg.br

Table S1 – Internal Solution used for the record of the action potential.

| Internal Solution | |
| --- | --- |
|  | Concentration (mM) |
| KCl | 20 |
| HEPES | 10 |
| KOH | 130 |
| Aspartate | 130 |
| MgCl_2_.6H_2_O | 2 |
| NaCl | 5 |

Note: For action potential recordings was used Tyrode as external solution.

Table S2 – External and internal Solutions used for the recording of potassium currents.

| **External Solution** | | **Internal Solution** | |
| --- | --- | --- | --- |
|  | **Concentration (mM)** |  | **Concentration (mM)** |
| NMDG | 140 | KCL | 140 |
| KCL | 5.6 | MgCl_2_.6H_2_O | 1 |
| MgCl_2_.6H_2_O | 0.5 | EGTA | 11 |
| CdCl_2_. H_2_O | 0.1 | HEPES | 10 |
| Glucose | 11 |  |  |
| HEPES | 10 |  |  |
| CaCl_2_.2H_2_O | 1.8 |  |  |

Table S3- Internal and external Solutions used for the calcium currents.

| **External Solution** | | **Internal Solution** | |
| --- | --- | --- | --- |
|  | **Concentration (mM)** |  | **Concentration (mM)** |
| TEA-Cl | 150 | CsCl | 140 |
| MgCl_2_.6H_2_O | 0.5 | MgCl_2_.6H_2_O | 1 |
| Glucose | 11 | HEPES | 10 |
| HEPES | 10 | EGTA | 10 |
| CaCl_2_.2H_2_O | 1.8 | Na_2_ATP | 5 |

Table S4 – External and internal Solutions used for the currents of sodium-calcium exchanger.

| **External Solution** | | **Internal Solution** | |
| --- | --- | --- | --- |
|  | **Concentration (mM)** |  | **Concentration (mM)** |
| NaOH | 145 | NaCl | 20 |
| Aspartate | 145 | MgCl_2_.6H_2_O | 1.19 |
| HEPES | 5 | HEPES | 10 |
| MgCl_2_.6H_2_O | 2 | TEA-Cl | 20 |
| CaCl_2_.2H_2_O | 1 | EGTA | 40 |
| BaCl_2_.2H_2_O | 2 | CsOH | 60 |
| Ouabain | 0.05 | CaCl_2_.2H_2_O | 33.8 |
| Nicardipine | 0.002 | Aspartate | 40 |

Table S5 – Cell contractility parameters WT and ARDKO at 7 months of age.

|  | Control | | | | |  | Isoproterenol | | | | |
| --- | --- | --- | --- | --- | --- | --- | --- | --- | --- | --- | --- |
|  | Wild Type (WT) | | ARDKO | | |  | Wild Type (WT) | | ARDKO | | |
|  | 1 Hz (n = 53) | 3 Hz (n = 46) | 1 Hz (n = 62) | | 3 Hz (n = 53) |  | 1 Hz (n = 35) | 3 Hz (n = 29) | 1 Hz (n = 32) | | 3 Hz (n = 23) |
| Diastolic length (µm) | 136.9 ± 25.4 | 135.5 ± 24.7 | 145.9 ± 31.3 | 143.0 ± 28.0 | |  | 133.8 ± 20.9 | 134.2 ± 21.0 | 137.2 ± 22.7 | 135.8 ± 26.5 | |
| Shortening fraction (%) | 9.5 ± 3.2 | 10.8 ± 3.9 | 6.4 ± 2.6^*^ | 7.3 ± 3.2^*^ | |  | 14.0 ± 3.6 | 13.5 ± 3.2 | 15.6 ± 3.0 | 15.6 ± 3.4^*^ | |
| Time to peak (ms) | 89.9 ± 15.0 | 79.7 ± 9.6^**^ | 88.5 ± 15.5 | 76.4 ± 12.5^**^ | |  | 76.9 ± 14.7 | 67.5 ± 7.6^**^ | 74.0 ± 12.3 | 67.0 ± 6.0^**^ | |
| dL/dt contraction (µm/s) | 296.5 ± 132.7 | 361.3 ± 166.0^**^ | 203.9 ± 103.7^*^ | 249.4 ± 128.6* | |  | 536.4 ± 192.0 | 524.1± 176.8 | 604.0 ± 148.9 | 629.9 ± 175.6* | |
| dL/dt relaxation (µm/s) | 270.7 ± 116.3 | 333.5 ± 157.5^**^ | 181.0 ± 109.8^*^ | 220.8 ± 129.2^*^ | |  | 491.1 ± 149.4 | 494.7± 167.3 | 526.1 ± 144.2 | 487.2 ± 121.3 | |
| 50% the relaxation (ms) | 41.2 ± 7.8 | 38.3 ± 6.3^*,**^ | 49.8 ± 16.0^*^ | 42.6 ± 11.9^**^ | |  | 37.2 ± 15.9 | 33.1 ± 13.1 | 35.9 ± 5.4 | 35.1 ± 5.6 | |
| 90% the relaxation (ms) | 82.4 ± 16.1 | 67.3 ± 10.2^*,**^ | 96.1 ± 33.5 * | 77.8 ± 21.4^*,**^ | |  | 68.2 ± 22.7 | 59.3 ± 15.9 | 67.6 ± 17.4 | 64.9 ± 15.0 | |

^*^ P < 0.05 compared to WT at the same frequency of stimulation within the same subgroup (Control or Isoproterenol); ^**^ P < 0.05 compared to the same strain (WT or ARDKO) at 1 Hz within the same subgroup. Cells obtained from 4 hearts of each group (Two-way ANOVA followed by Bonferroni).

Table S6 – Action potential parameters of WT and ARDKO cardiomyocytes at 7 months of age.

|  | Wild Type (WT) | |  | α2a/α2c KO (ARDKO) | |
| --- | --- | --- | --- | --- | --- |
|  | 1 Hz (n = 34) | 3 Hz (n = 24) |  | 1 Hz (n = 32) | 3 Hz (n = 26) |
| Resting potential (mV) | -76.2 ± 2.6 | -76.9 ± 2.4 |  | -75.0 ± 1.8 | -75.4 ± 2.3 |
| Overshoot (mV) | 34.9 ± 7.7 | 32.5 ± 7.5 |  | 36.8 ± 8.0 | 32.4 ± 9.1 |
| Amplitude (mV) | 111.2 ± 9.1 | 109.3 ± 8.9 |  | 111.9 ± 8.8 | 107.8 ± 10.5 |
| + dV/dt (V/s) | 167.4 ± 49.3 | 149.1 ± 40.4 |  | 166.5 ± 59.7 | 131.2 ± 53.8 |
| -dV/dt (V/s) | -22.0 ± 12.2 | -16.9 ± 6.6^**^ |  | -13.1 ± 6.3^*^ | -10.0 ± 7.7^*,**^ |
| 10% Repolarization (ms) | 0.8 ± 0.3 | 1.1 ± 0.5 |  | 1.3 ± 0.4 | 2.2 ± 2.0^*,**^ |
| 50% Repolarization (ms) | 7.2 ± 4.3 | 9.6 ± 6.9 |  | 14.8 ± 7.7^*^ | 22.3 ± 15.6^*,**^ |
| 90% Repolarization (ms) | 136.5 ± 86.0 | 124.1 ± 62.6 |  | 205.5 ± 65.7^*^ | 179.6 ± 55.3^*^ |

^*^ P < 0.05 compared to WT at the same frequency of stimulation; ^**^ P < 0.05 compared to 1 Hz within the same strain (WT or ARDKO). Cells obtained from 4 hearts of each group (Two-way ANOVA followed by Bonferroni).

Table S7 – Cell contractility parameters WT and ARDKO at 3 months of age.

|  | Control | | | | |  | Isoproterenol | | | | |
| --- | --- | --- | --- | --- | --- | --- | --- | --- | --- | --- | --- |
|  | Wild Type (WT) | | ARDKO | | |  | Wild Type (WT) | | ARDKO | | |
|  | 1 Hz (n = 22) | 3 Hz (n = 22) | 1 Hz (n = 23) | | 3 Hz (n = 19) |  | 1 Hz (n = 18) | 3 Hz (n = 15) | 1 Hz (n = 19) | | 3 Hz (n = 19) |
| Diastolic length (µm) | 150.1 ± 26.6 | 150.1 ± 24.9 | 121.0 ± 17.4 | 117.8 ± 17.8 | |  | 138.3 ± 17.2 | 136.5 ± 17.7 | 128.4 ± 18.6 | 127.3 ± 18.4 | |
| Shortening fraction (%) | 7.5 ± 3.4 | 10.6 ± 3.5^**^ | 8.6 ± 3.1 | 11.4 ± 4.0^**^ | |  | 10.4 ± 4.8 | 12.0 ± 4.6 | 12.2 ± 3.1 | 12.8 ± 3.0 | |
| Time to peak (ms) | 84.5 ± 14.4 | 77.9 ± 10.1 | 83.01 ± 16.2 | 80.3 ± 19.7 | |  | 67.3 ± 9.3 | 67.3 ± 5.4 | 69.6 ± 12.2 | 64.9 ± 8.0 | |
| dL/dt contraction (µm/s) | 243.8 ± 94.6 | 378.4 ± 133.6^**^ | 226.8 ± 86.5 | 319.7 ± 114.3^**^ | |  | 429.1 ± 255.7 | 474.5 ± 224.6 | 437.2 ± 149.9 | 477.9 ± 137.5 | |
| dL/dt relaxation (µm/s) | 203.3 ± 76.4 | 341.9 ± 112.4^**^ | 202.7 ± 94.9 | 321.2 ± 124.5^**^ | |  | 388.5 ± 222.5 | 444.8 ± 183.9 | 426.2 ± 139.7 | 472.2 ± 136.2 | |
| 50% of relaxation (ms) | 43.7 ± 5.8 | 35.7 ± 5.7^**^ | 41.9 ± 10.2 | 32.4 ± 5.9^**^ | |  | 31.8 ± 7.4 | 27.2 ± 3.9^**^ | 28.6 ± 4.2 | 26.1 ± 3.9 | |
| 90% of relaxation (ms) | 104.1 ± 19.0 | 74.3 ± 14.9^**^ | 105.7 ± 29.7 | 67.8 ± 15.9^**^ | |  | 74.4 ± 24.6 | 57.8 ± 15.3^**^ | 65.9 ± 17.5 | 57.3 ± 16.5 | |

^**^ P < 0.05 compared to the same strain (WT or ARDKO) at 1 Hz within the same subgroup. Cells obtained from 4 hearts of each group (Two-way ANOVA followed by Bonferroni).

Table S8 – Action potential parameters of cardiomyocytes WT and ARDKO at 3 months of age.

|  | Wild Type (WT) | |  | α2a/α2c KO (ARDKO) | |
| --- | --- | --- | --- | --- | --- |
|  | 1 Hz (n = 15) | 3 Hz (n = 13) |  | 1 Hz (n = 16) | 3 Hz (n = 12) |
| Resting potential (mV) | -74.4 ± 2.5 | -74.6 ± 2.4 |  | -75.2 ± 2.2 | -75.0 ± 2.0 |
| Overshoot (mV) | 36.2 ± 5.8 | 34.2 ± 5.56 |  | 39.4 ± 5.6 | 37.1 ± 5.9 |
| Amplitude (mV) | 110.7 ± 7.3 | 108.2 ± 7.4 |  | 112.4 ± 5.4 | 112.8 ± 6.6 |
| + dV/dt (V/s) | 122.4 ± 31.9 | 108.5 ± 28.9 |  | 128.6 ± 50.8 | 102.7 ± 34.0 |
| -dV/dt (V/s) | -21.4 ± 6.5 | -19.8 ± 6.1 |  | -23.9 ± 25.2 | -15.3 ± 6.4 |
| 10% Repolarization (ms) | 0.9 ± 0.3 | 1.0 ± 0.3 |  | 1.1 ± 0.5 | 1.2 ± 0.6 |
| 50% Repolarization (ms) | 5.2 ± 2.9 | 5.5 ± 3.1 |  | 7.5 ± 5.0 | 8.9 ± 6.1 |
| 90% Repolarization (ms) | 61.1 ± 29.9 | 68.3 ± 35.2 |  | 54.1 ± 31.1 | 89.4 ± 33.5^**^ |

^**^ P < 0.05 compared to 1 Hz within the same strain (WT or ARDKO). Cells obtained from 4 hearts of each group (Two-way ANOVA followed by Bonferroni).

Table S9 – Computational model parameters adjusted for ARDKO simulations. See Wescott et al., 2016 for all other parameters.

| **Parameter** | **Description** | **Percent of WT** |
| --- | --- | --- |
| *g_ktof_* | Conductance for fast outward K^+^ current | 50 |
| *g_ktos_* | Conductance for slow outward K^+^ current | 70 |
| *P_dhpr_* | LCC permeability | 70 |
| *I_ncx1_* | Maximal NCX current density | 150 |
| *A_p_* | SERCA density | 120 |


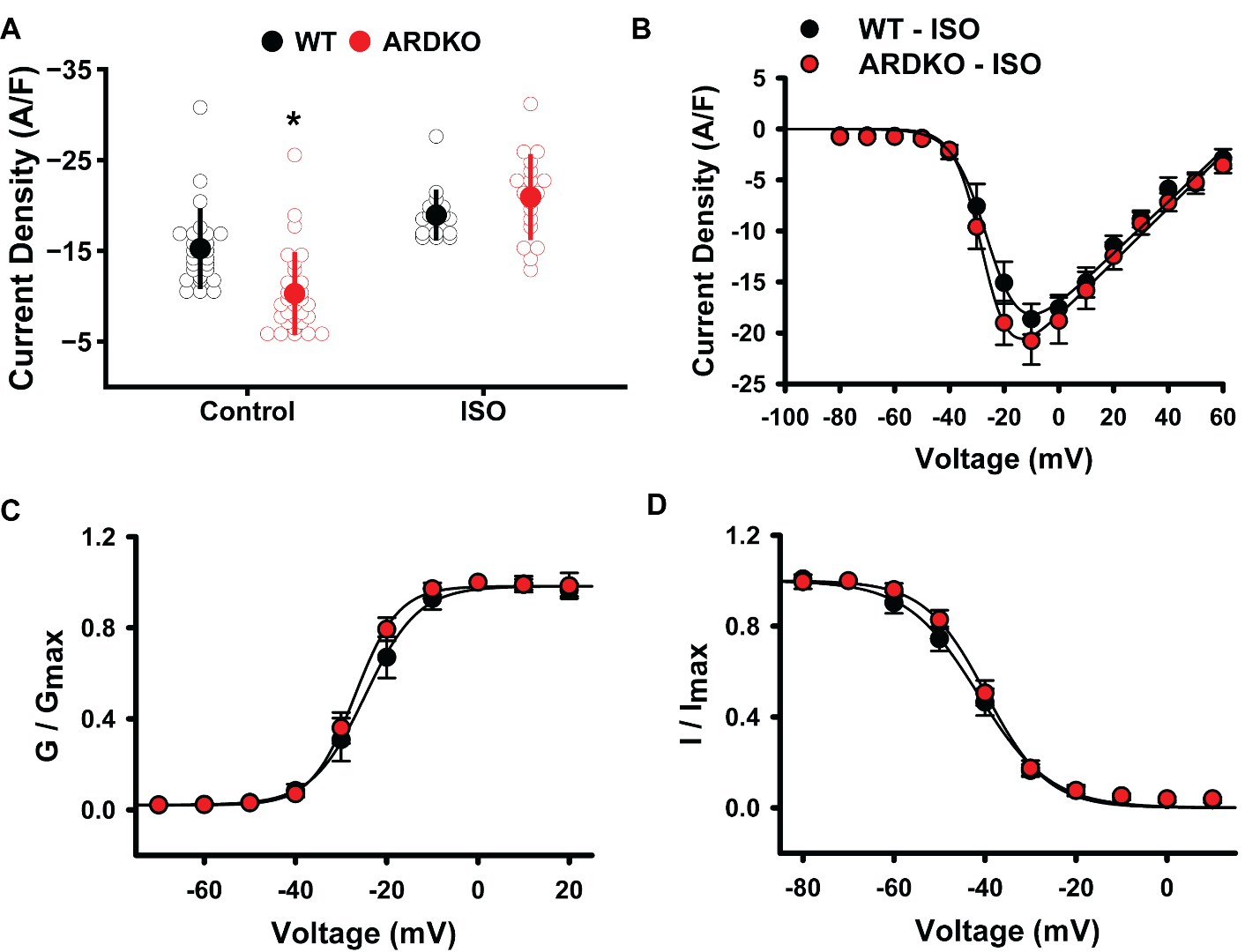


**Figure S1.** ARDKO myocytes have reduced calcium current density (**A**), however that difference is abolished in the presence of isoproterenol (100 nM). When stimulated with Isoproterenol, ARDKO myocytes have no significative changes in current density (**B**), activation (**C**) and inactivation (**D**) voltage-dependence (WT n = 16 cells / 3 hearts; ARDKO n = 18 cells / 3 hearts). * p < 0.05 when compared to WT without Isoproterenol (Control).


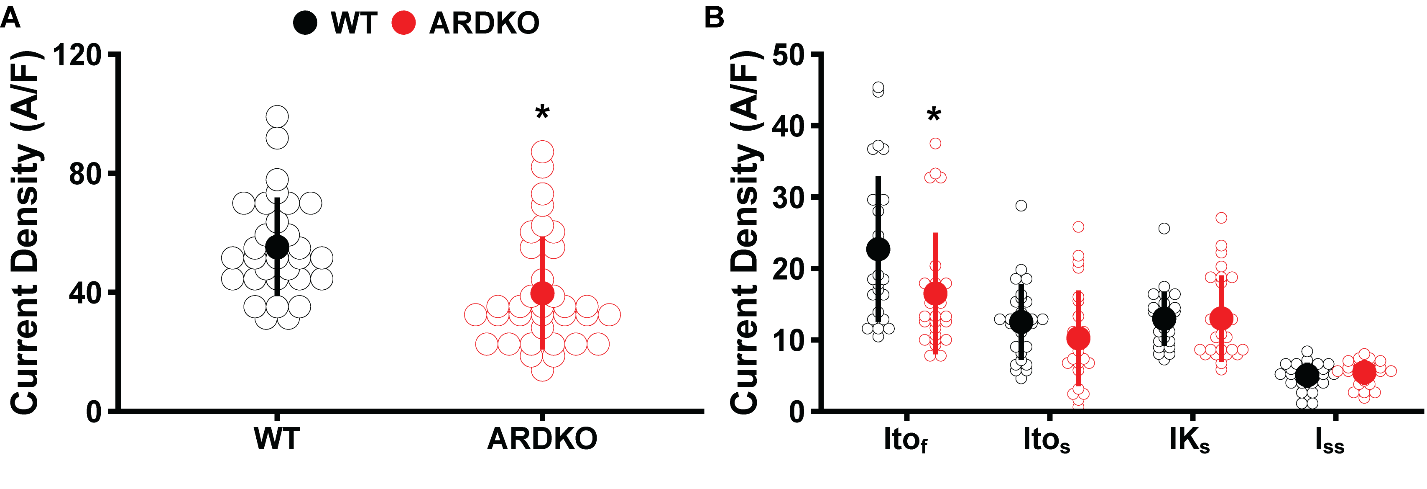


**Figure S2.** ARDKO myocytes have a reduction in voltage-gated outward potassium current (**A**). Such difference, however, is mostly related to a down-regulation of the fast transient outward current component (I_tof_) (**B**) (WT n = 31 cells / 4 hearts; ARDKO n = 34 cells / 4 hearts). * p < 0.05 when compared to WT.


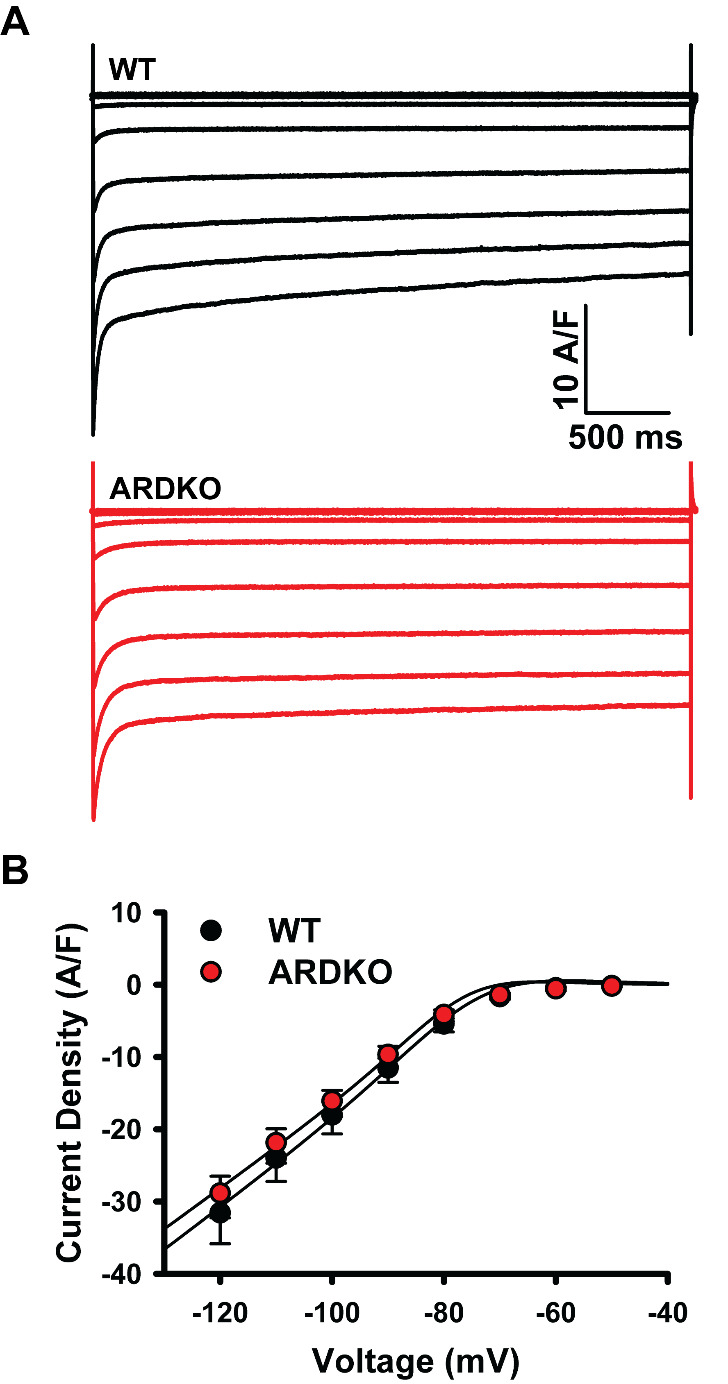


**Figure S3.** Inward rectifier potassium currents are not involved in the cardiac action potential remodeling observed in ARDKO myocytes. Panel **A** shows the representative traces for Inward rectifier currents recorded from WT (black) and ARDKO (red) cells. No changes were observed in the current density (**B**). (WT n = 31 cells / 4 hearts; ARDKO n = 34 cells / 4 hearts).


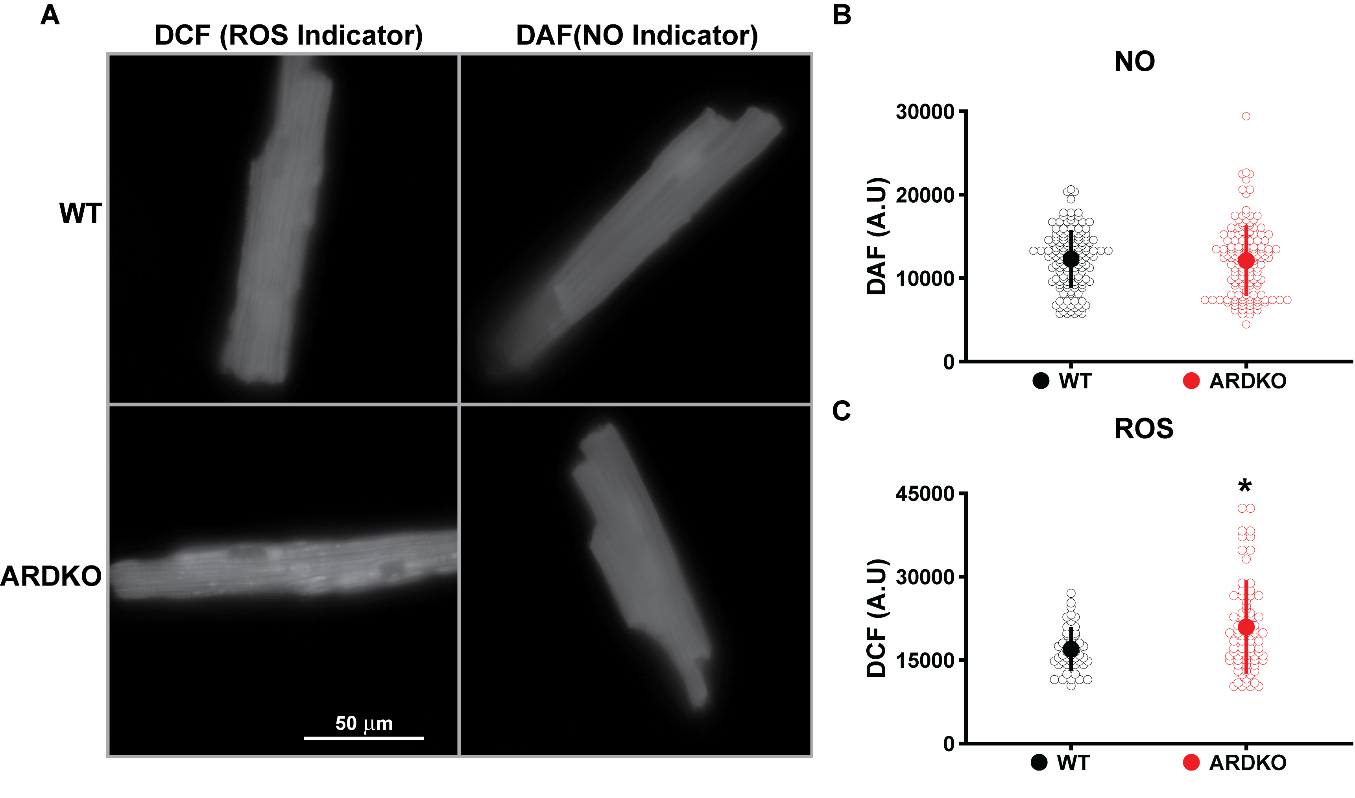


**Figure S4.** ARDKO myocytes have increased availability of reactive oxygen species (ROS). Panel A shows representative fluorescence images from WT and ARDKO myocytes using a NO indicator (DAF) and ROS indicator (DCF). No changes in NO levels were observed (**B**) (WT n = 151 cells / 5 hearts; ARDKO n = 147 cells / 5 hearts), however, a higher level of ROS was measured in ARDKO cardiac cells (**C**) (WT n = 47 cells / 5 hearts; ARDKO n = 78 cells / 5 hearts). * p < 0.05 when compared to WT (Student’s t-test).


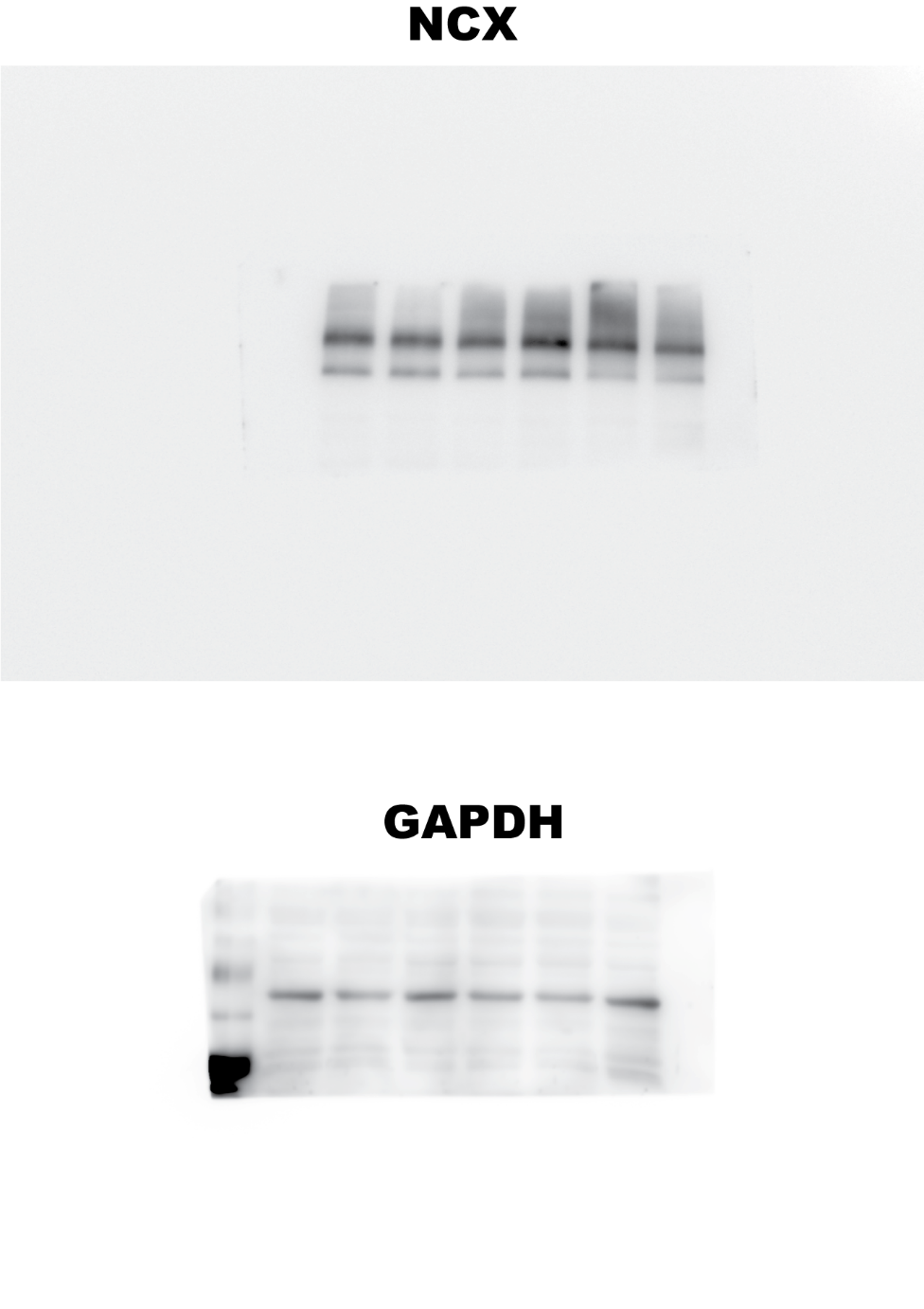


**Figure S5.** Full-length images from Western blot for NCX (upper image) and GAPDH (lower image) used for the inset of Figure 5, panel B.
